# Relief of ParB autoinhibition by *parS* DNA catalysis and recycling of ParB by CTP hydrolysis promote bacterial centromere assembly

**DOI:** 10.1126/sciadv.abj2854

**Published:** 2021-10-06

**Authors:** Hammam Antar, Young-Min Soh, Stefano Zamuner, Florian P. Bock, Anna Anchimiuk, Paolo De Los Rios, Stephan Gruber

**Affiliations:** 1 Department of Fundamental Microbiology (DMF), Faculty of Biology and Medicine (FBM), University of Lausanne, 1015 Lausanne, Switzerland.; 2 Laboratory of Statistical Biophysics, Institute of Physics, School of Basic Sciences and Institute of Bioengineering, School of Life Sciences, Ecole Polytechnique Fédérale de Lausanne (EPFL), 1015 Lausanne, Switzerland.

## Abstract

Three-component ParABS systems are widely distributed factors for plasmid partitioning and chromosome segregation in bacteria. ParB acts as adaptor protein between the 16–base pair centromeric *parS* DNA sequences and the DNA segregation proteins ParA and Smc (structural maintenance of chromosomes). Upon cytidine triphosphate (CTP) and *parS* DNA binding, ParB dimers form DNA clamps that spread onto *parS*-flanking DNA by sliding, thus assembling the so-called partition complex. We show here that CTP hydrolysis is essential for efficient chromosome segregation by ParABS but largely dispensable for Smc recruitment. Our results suggest that CTP hydrolysis contributes to partition complex assembly via two mechanisms. It promotes ParB unloading from DNA to limit the extent of ParB spreading, and it recycles off-target ParB clamps to allow for *parS* retargeting, together superconcentrating ParB near *parS*. We also propose a model for clamp closure involving a steric clash when binding ParB protomers to opposing *parS* half sites.

## Teaser

CTP binding and hydrolysis concentrate ParB at parS centromeres to mediate faithful chromosome segregation in bacteria.

## INTRODUCTION

Faithful transmission of genetic material from one generation to the next is a prerequisite for survival and propagation. Chromosome segregation is spatially and temporally coordinated with DNA replication, cell division, and cell growth to maintain genome integrity and cell architecture over multiple generations. In bacteria, three-component ParABS systems promote faithful and efficient chromosome segregation (*1*–*3*). They are also important regulators of cell physiology with mutations leading to diverse and pleiotropic phenotypes in addition to chromosome partitioning errors. These phenotypes include defects in the control of DNA replication, chromosome organization, cell division, gene expression, motility, sporulation, and competence development (*1*, *4*–*14*).

ParABS systems are present on most bacterial genomes and are also frequently encoded by low-copy number plasmids (*15*). They comprise 16–base pair (bp) DNA sequence elements, called *parS* sites, the DNA binding protein ParB, and the adenosine triphosphate (ATP)–hydrolyzing protein ParA. ParB proteins and *parS* sites together form the partition complex that serves as chromosome organizing center (“*parS* centromere”) (*3*, *16*). Partition complexes are thought to follow ParA protein gradients on the bacterial chromosome to become equidistantly positioned within the cell (*12*, *17*, *18*). They stimulate ATP hydrolysis by ParA thus converting DNA-bound ParA dimers into ParA monomers that dissociate from chromosomal DNA (*5*, *19*–*21*). Using the same “diffusion-ratchet” mechanism, ParABS is thought to promote plasmid partitioning (*18*, *22*). The *parS* centromere also instructs another active DNA segregation mechanism, the Smc (structural maintenance of chromosomes) DNA loop extrusion motor. Starting from *parS* centromeres, the Smc complex aligns the two chromosome arms, helping to segregate nascent sister chromosomes (*6*, *23*–*26*). ParABS furthermore regulates the initiation of DNA replication via the initiator protein DnaA in *Bacillus subtilis*. Inability to convert ParA ATP dimers into monomers (e.g., in a Δ*parB* mutant) leads to overinitiation of DNA replication (*4*) and, as a consequence, also blocks sporulation (*27*).

*parS* sequences are positioned near the replication origin, often in multiple copies scattered over a more or less wide replication origin region (<1 Mb) (*15*). ParB proteins accumulate in high numbers near a given *parS* site, leading to the formation of distinctive protein clusters in the cell (*28*–*30*). A related and essential feature of ParB is the ability to occupy not only the *parS* recognition sequence itself but also flanking DNA sequences. The spreading of ParB was first observed indirectly by its effects on plasmid supercoiling and silencing of *parS* proximal genes (*31*, *32*). Chromosomal ParB is enriched in regions ranging from few kilobases up to ~15 kb around *parS* sites in chromatin immunoprecipitation (ChIP) profiles (*28*, *33*, *34*). We and others have recently found that ParB spreading requires the binding of the unusual cofactor cytidine triphosphate (CTP) by ParB (*16*, *35*–*37*). On the basis of a nucleotide-ParB costructure, we have proposed that ParB dimers form DNA sliding clamps that entrap chromosomal DNA in a *parS-*catalyzed closure reaction to then slide onto *parS*-flanking DNA (*37*).

ParB proteins comprise three globular domains. The N domain forms a conserved binding pocket for the ribonucleotide CTP (*36*, *37*). CTP binding [dissociation constant ~ 10 μM] (*35*, *37*) is expected to be nearly saturated under standard physiological conditions (~100 to 200 μM CTP). Upon contact with *parS* DNA, open ParB dimers convert into closed clamps by the CTP-bound N domains forming interlocking dimers. N-gate closure also occurs in the absence of *parS* DNA but is slow. The *parS*-specific recognition of DNA originates from a helix-turn-helix (HTH) motif located in the ParB M domain (*38*). The C domain supports ligand-independent ParB dimerization and sequence-unspecific DNA binding (*39*, *40*). CTP hydrolysis does not seem to be required for any step of ParB targeting and sliding in vitro (*35*, *37*).

Here, we aimed to get a better understanding of the physiological role of CTP hydrolysis by generating ParB mutants in *B. subtilis*. We identified two CTP hydrolysis-defective mutants of ParB, which retained the ability to bind CTP, to close the N-gate and to load onto *parS* DNA in vitro. We showed that CTP hydrolysis is essential for normal ParB focus formation in vivo. It serves two main functions by recovering CTP-locked ParB clamps trapped off the chromosomes and by restricting the extent of ParB spreading through DNA unloading. Despite the aberrant localization, CTP hydrolysis–defective ParB mutants supported normal sporulation and Smc recruitment, but the mutant ParABS systems were unable to support chromosome segregation in the absence of Smc. Moreover, we present a model for *parS*-catalyzed ParB DNA loading based on *parS*-mediated relief of ParB autoinhibition.

## RESULTS

Efficient chromosomal loading of ParB clamps requires the cofactor CTP and the catalyst *parS* DNA. CTP hydrolysis is thought to promote the reverse reaction, ParB clamp unloading. Here, we investigate the molecular mechanisms underlying these reactions and the physiological consequences of blocking CTP hydrolysis.

### Intra- and intermolecular tethering of N and M domains

**Fig. 1.**
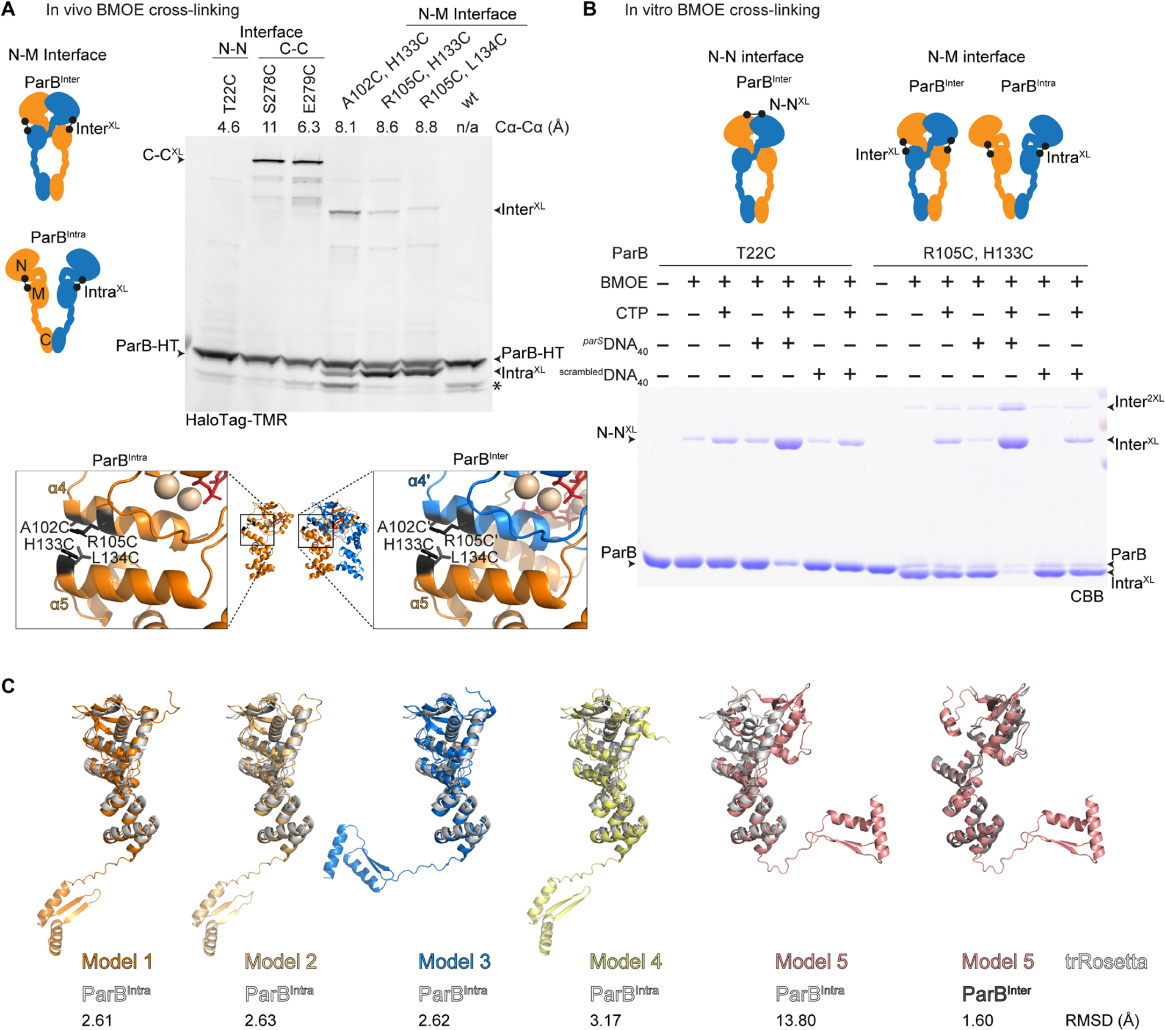
Intra- and intermolecular tethering of ParB domains. (**A**) In vivo BMOE cross-linking using ParB cysteine variants harboring a HaloTag (“HT”). “Intra^XL^” and “Inter^XL^” denote cross-links formed between N and M domains of the same and different protomers, respectively. Cα-Cα distances on the ParB^Inter^ dimer (PDB: 6SDK and 5NOC) are denoted. The asterisk indicates a putative degradation product of ParB-HT. Structure inserts denote the cysteines on the ParB^Inter^ dimer structure (PDB: 6SDK) (right) and on a manually created ParB^Intra^ model (left) (fig. S2C). wt, wild type; n/a, not applicable. (**B**) In vitro cross-linking of ParB cysteine variants (at a concentration of 10 μM). The cross-linking was performed with CTP (1 mM), 40-bp *parS*, or *parS*-free DNA (*^parS^*DNA_40_ or ^scrambled^DNA_40,_ respectively; 1 μM). Protein species were visualized on SDS-PAGE by Coomassie Brilliant Blue (CBB) staining. For quantification, see fig. S2A. (**C**) ParB folding was predicted by *Robetta* with *B. subtilis* ParB as input (UniProt: P26497). The top five hits were superimposed with the ParB^Intra^ model. Four superimpositions (1 to 4) displayed close resemblance. The remaining fold (5; left) resulted in a good match only when superimposed with ParB^Inter^ (PDB: 6SDK) (5; right). Root mean square displacements (RMSDs; in angstroms) are shown as structural similarity indicator.

In the crystal structure of a cytidine 5′-diphosphate (CDP)–bound *B. subtilis* ParB dimer, the N domain of a given ParB chain closely associates with the M domain of the partner chain [Protein Data Bank (PDB): 6SDK; Fig. 1A and fig. S1A] (*37*). An equivalent domain swapping organization has recently been reported for a ParB paralog, the *B. subtilis* protein Noc (PDB: 7NG0) (*41*), and has previously been noted in the CTP-bound dimer of *Myxococcus xanthus* PadC (PDB: 6RYK) (*36*). This suggests that domain swapping is not a crystal packing artifact but a conserved feature of ParB and ParB-like proteins.

To test whether ParB adopts the domain swapping organization in vivo, we performed site-specific chemical cross-linking at the N-M interface using strains with a *halotag* (*HT*) fused to *parB* at the endogenous gene locus for the detection and quantification of cross-linked species (Fig. 1A) (*37*). Residues on helix α4 in the N domain (A102C and R105C) and on helix α5 in the M domain (H133C and L134C) were selected for cysteine mutagenesis (Fig. 1A). One of the four cysteine pairs (A102C/L134C) was omitted from further analysis, as it exhibited a sporulation defect, being indicative of nonfunctional protein (*42*). The three other pairs did not grossly affect ParB function as indicated by the absence of a noticeable sporulation defect and by normal growth of the double cysteine mutants in a sensitized background harboring the *smc-pk3* allele (fig. S1B) (*24*). If intermolecular N-M tethering does occur, then cross-linking with the thiol-specific compound bismaleimidoethane (BMOE) would generate a dimeric ParB-HT species that is expected to migrate more slowly through an SDS–polyacrylamide gel electrophoresis (PAGE) gel. We indeed observed such a slowly migrating species (Inter^XL^) with all tested cysteine combinations (Fig. 1A and fig. S1C). The efficiency of intermolecular cross-linking was low and slightly higher only with one of the three cysteine pairs (A102C/H133C). We also detected a more prominent, faster migrating cross-linked ParB-HT product, which we interpreted as a species derived from intramolecular cross-linking of N and M domains (Intra^XL^) (Fig. 1A).

This experiment suggested that N-M tethering occurs in two ways: within a protomer (“ParB^Intra^”) and between two protomers (“ParB^Inter^”) (Fig. 1, A and B). To elucidate how the domain swapping configuration is established, we next purified ParB(R105C, H133C) protein for in vitro cross-linking experiments. In the absence of ligands, BMOE cross-linking robustly produced the faster migrating species (Intra^XL^) and very little of two slowly migrating species (Inter^XL^ and Inter^2XL^), corresponding to single and double cross-linked ParB dimers (Fig. 1B and fig. S2A). We conclude that most or all ParB proteins displayed the ParB^Intra^ configuration before ligand binding. Addition of CTP or 40-bp *parS* DNA (“*^parS^*DNA_40_”) alone did not alter the cross-linking pattern noticeably while addition of both strongly increased the abundance of the two dimeric species and in turn almost eliminated the intramolecular cross-linking. Similar results were obtained with ParB(A102C, H133C) (fig. S2B). The requirement for both ligands mimicked what was observed for N-gate closure by T22C cross-linking (Fig. 1B) (*37*). N-gate closure thus goes along with the conversion of ParB^Intra^ into ParB^Inter^ conformations.

### A mechanism for *parS*-catalyzed ParB loading based on ParB autoinhibition

The closed ParB^Inter^ state is thermodynamically favorable in the presence of CTP (and CTPγS but not CDP) (*37*). The conversion of CTP-bound ParB^Intra^ to ParB^Inter^, however, is slow without *parS* DNA (i.e., it is a kinetically inhibited reaction) (fig. S2C) (*37*). What inhibits the reaction in the absence of *parS* DNA is unclear. Disengagement of intramolecular N-M tethers in both ParB^Intra^ protomers is presumably a prerequisite for forming the closed N-gate built from two ParB^Inter^ protomers. We thus wondered whether the intramolecular N-M tether inhibits N-gate closure. If so, then ParB^Intra^ would correspond to an autoinhibited form of ParB. Stochastic N-M disengagement is likely inefficient because of rapid reengagement of the closely linked N and M domains, even in case of limited N-M tether stability.

**Fig. 2.**
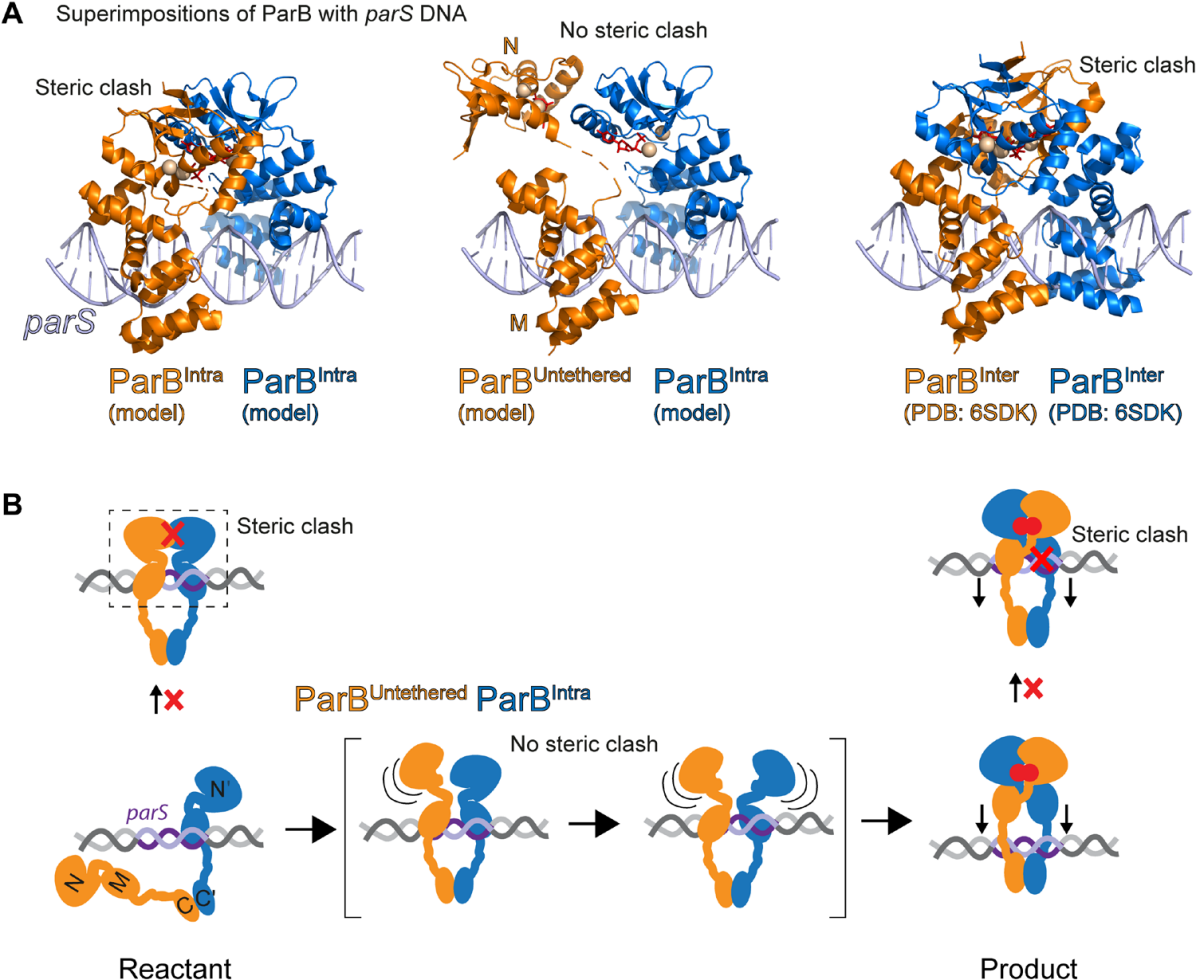
Steric hindrance of *parS* DNA binding by ParB^Intra^ dimers and ParB^Inter^ dimers. (**A**) Superimposition of ParB chains with *parS* DNA. Structural models of the ParB^Intra^ state were manually created from the ParB^Inter^ structure (PDB: 6SDK) (fig. S3A) or computationally predicted (Fig. 1C). Binding to *parS* DNA was modeled by superimposition of ParB^Intra^ (left) and ParB^Inter^ with a *H. pylori* ParB-*parS* cocrystal structure (PDB: 4UMK) using the HTH motif as a guide for structural alignment yielding a steric clash of the N domains. A ParB^Untethered^ model was generated by manually detaching the N domain from the M domain. Superimposition with *parS* DNA (middle) was possible without steric hindrance. (**B**) A tentative model for *parS*-catalyzed N-gate closure. ParB dimers fail to bind to both *parS* half sites simultaneously due to steric clash. Only when the N domain in at least one ParB protomer is partially unfolded or untethered from the M domain does the binding to both *parS* half sites become feasible. The interlocking of the two ParB protomers (ParB^Inter^ N-M) recreates the steric clash when binding to *parS* DNA, thus releasing the closed ParB dimer from *parS* DNA.

To uncover how this ParB autoinhibition might be overcome by *parS* DNA binding, we generated a structural model of ParB^Intra^ by manually reconnecting the chains at the N to M junction of the CDP-bound ParB dimer (PDB: 6SDK; fig. S3A). We also computationally predicted ParB structure de novo using trRosetta (*43*). One of the top five hits closely resembled the ParB^Inter^ structure, while the other four matched well with the manually created ParB^Intra^ model, providing unbiased and independent support for the existence of the two states (Fig. 1C). Next, we superimposed two ParB^Intra^ chains with opposing halves of a *parS* site (PDB: 4UMK), using the HTH in the M domain as guide for structural alignment. We found that a single ParB^Intra^ protomer can readily accommodate a *parS* half site, but two ParB^Intra^ protomers clash with one another when bound to opposing half sites, indicating that a ParB^Intra^ dimer fails to strongly bind *parS* DNA (Fig. 2A). However, when the N domain was manually detached from the M domain (“ParB^Untethered^”) in at least one of the two protomers, then binding of both HTH motifs to the half sites of a given *parS* DNA sequence becomes feasible without steric clash (Fig. 2A). Thus, we propose that *parS* DNA catalyzes N-gate closure by preventing N-M reengagement and selecting and stabilizing a ParB^Untethered^ state (Fig. 2B). With one protomer being kept in this—otherwise thermodynamically unfavorable—state, N-gate closure may proceed efficiently as soon as the other protomer stochastically happens to adopt the same state. Consistent with this hypothesis, the N domain of at least one of the two *parS*-bound ParB chains has been observed in a partially unfolded state in available ParB-*parS* cocrystal structures (PDB: 4UMK) (*38*) (PDB: 6T1F) (*44*). Accordingly, open ParB dimers exist as an ensemble of states with protomers in ParB^Intra^ and ParB^Untethered^ conformations, while closed ParB dimers are built from ParB^Inter^ protomers.

A prediction from this Brownian-ratchet reaction scheme is that the steric clash between two ParB^Intra^ protomers at *parS* DNA is crucial for the stimulation of N-gate closure by *parS* DNA. Aiming to artificially eliminate the steric clash, we next inserted an increasing number of spacer nucleotides (adding 1 to 8 bp) between the two *parS* half sites in *^parS^*DNA_40_. While we did not observe notable changes in ParB affinity by anisotropy measurements with fluorescently labeled DNA_40_, we found that all altered *parS* sites failed to stimulate CTP hydrolysis, an indirect readout for the efficiency of N-gate closure. DNA molecules with intermediate spacer lengths (2 to 5 bp) not only failed to stimulate but also actually inhibited the reaction when present at stoichiometric amounts (fig. S3B). These observations are consistent with the idea of a steric clash between *parS*-bound ParB^Intra^ protomers being the basis of *parS*-catalyzed ParB DNA loading. Notably, ParB binding to isolated *parS* half sites has been observed in ChIP sequencing (ChIP-seq) profiles for *P. aeruginosa* ParB, particularly upon ParB overexpression (*45*), and possibly also in other organisms (*46*).

### CTP hydrolysis–defective ParB mutants

**Fig. 3.**
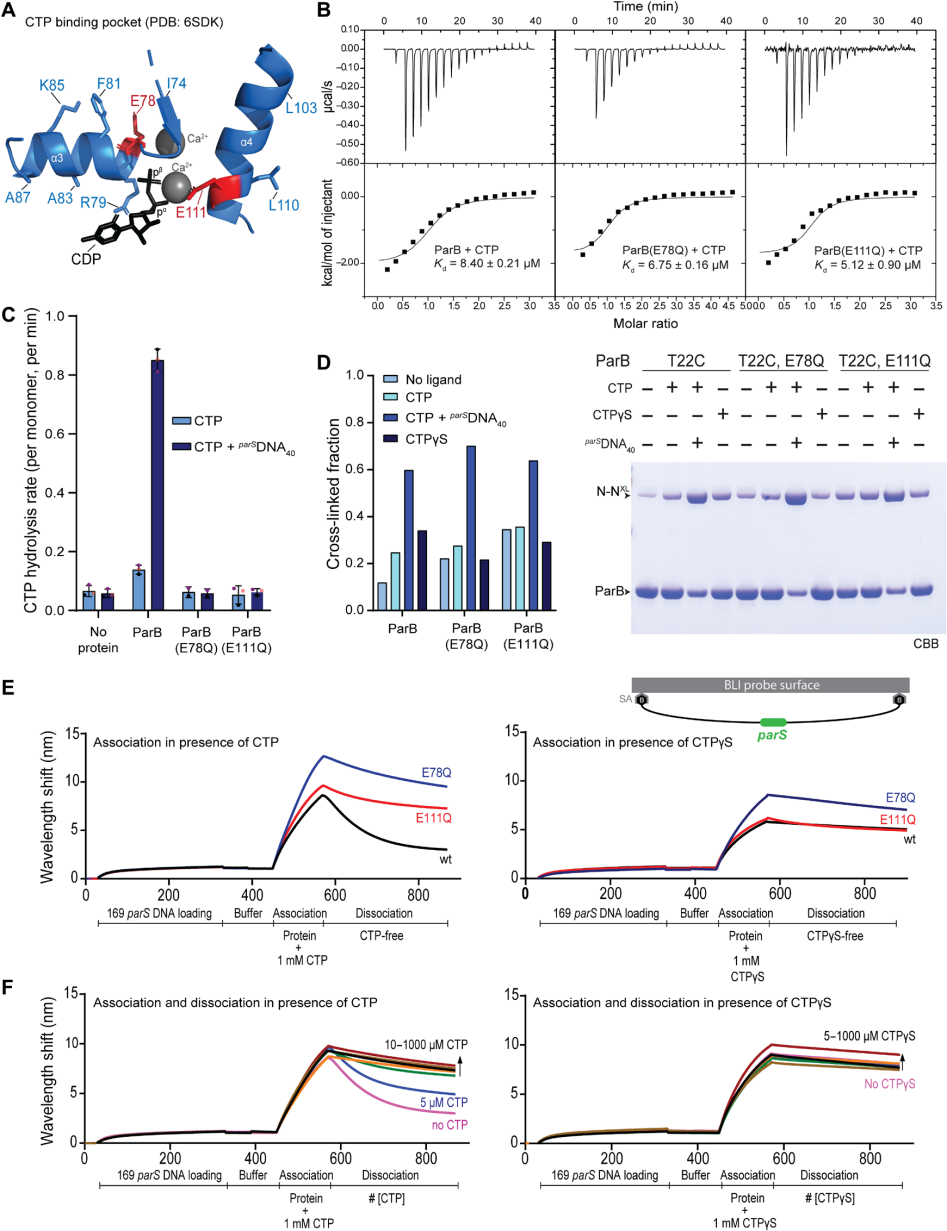
Hydrolysis-defective mutants of ParB. (**A**) The CTP binding pocket found in a ParB-CDP cocrystal structure (PDB: 6SDK) in cartoon representation. Conserved residues are shown in stick representation. Candidate catalytic residues, glutamate at positions 78 and 111, are highlighted in red colors. (**B**) ParB-CTP affinity measurements by ITC. The *K*_d_ from a typical experiment is given. For details, see Materials and Methods. (**C**) CTP hydrolysis rates assayed by colorimetric detection of inorganic phosphate. Ten micromolars of ParB was incubated with 1 mM CTP with or without 1 μM *^parS^*DNA_40_. Mean values and SD from three repeat measurements are reported. Individual data points are shown as dots. (**D**) ParB N-gate closure measured by in vitro BMOE cross-linking of ParB(T22C). Final reactions contained 10 μM ParB with or without 1 mM nucleotide triphosphate (NTP) and 1 μM *^parS^*DNA_40_. N-N^XL^ denotes cross-linked species. Protein species were separated by SDS-PAGE and visualized by CBB staining (right). (**E**) DNA loading of ParB (1 μM) on biotin-immobilized 169-bp *parS* DNA measured by BLI with CTP and CTPγS (left and right, respectively; 1 mM). During dissociation, an equivalent buffer lacking ParB protein and nucleotide was applied. (**F**) Dissociation of wild-type ParB from *parS* DNA in the presence of nucleotide. Same as in (E) but with increasing concentrations of CTP (left) and CTPγS (right) present during dissociation.

We next focused on the reverse reaction, i.e., ParB clamp opening, which is presumably supported by CTP hydrolysis. The ParB cytidine triphosphatase (CTPase) is a recently found member of a larger family of proteins with diverse enzymatic activities and cofactors (*37*, *47*). Little is yet known about the mechanism of ParB CTP hydrolysis. In ATP and guanosine triphosphate hydrolysis, a water molecule attacks the γ-phosphate moiety to hydrolyze the scissile bond between β- and γ-phosphates. The water molecule is usually activated for nucleophilic attack by an acidic residue, as, for example, found in Walker B motifs. To identify residues with equivalent functions in ParB, we biochemically characterized ParB proteins harboring mutations in selected active site residues. The desired mutants were expected to be defective in CTP hydrolysis but retain their ability to bind CTP, engage the N domains, and entrap *parS* DNA. The ParB-CDP cocrystal structure (PDB: 6SDK) highlighted two glutamate residues (at positions 78 and 111) at the CTP binding pocket (Fig. 3A) (*37*). These residues belong to widely conserved sequence motifs (GE_78_RRY/F and E_111_NLQR), but they are absent from *M. xanthus* PadC protein that binds CTP but does not hydrolyze it (with F348 in PadC corresponding to *B. subtilis* ParB E78 and PadC lacking the ENLQR motif altogether) (fig. S5A) (*36*). Examining the PadC-CTP interaction map confirmed that residue F348 is suitably positioned next to the CTP γ-phosphate (fig. S5B). The glutamate residues were chosen as candidates for further analysis and replaced separately by glutamine (Q). Substitutions for alanine or histidine produced similar initial results but were deemed more intrusive and thus omitted from further investigations (fig. S7C). The E78Q and E111Q mutant proteins were recombinantly expressed and purified. We assayed for CTP binding affinity, for the rate of CTP hydrolysis, for the efficiency of N-gate closure, and for ParB loading onto *parS* DNA. Our results showed that the E78Q and E111Q mutants failed to display appreciable levels of CTP hydrolysis as measured by the release of inorganic phosphate, while a wild-type control protein hydrolyzed CTP with a basal rate of approximately 0.15 min^−1^, which was stimulated by the presence of *parS* DNA to about 1 min^−1^, comparable with published results (Fig. 3C) (*37*). All three proteins bound CTP with similar affinity (*K*_d_ ~ 5 to 8 μM) as judged by isothermal titration calorimetry (ITC; Fig. 3B), and they had roughly comparable efficiencies of N-gate closure with CTP and *parS* DNA as measured by T22C cross-linking (Fig. 3D). They also loaded onto *parS* DNA in the presence of CTP based on biolayer interferometry (BLI) (Fig. 3E). Briefly, BLI allowed for the immobilization of a double biotin-labeled 169-bp *parS* DNA fragment on a streptavidin-coated biosensor tip (*35*). Binding of ligands, including ParB protein, is inferred from a wavelength shift in the reflected light. Association with *parS* DNA appeared normal or even slightly improved with the EQ mutants in the presence of CTP or the nonhydrolyzable analog CTPγS. When shifting the biosensor from the CTP-containing loading buffer to a CTP-free dissociation buffer, wild-type ParB protein was released from DNA with an estimated apparent rate of about 0.4 min^−1^, while the two EQ mutants displayed a significantly longer residence time on DNA. These differences in the dissociation rates between wild-type and EQ proteins were eliminated when CTPγS was used instead of CTP during loading. Notably, we observed that the presence of CTP in the dissociation buffer significantly reduced the off-rate of wild-type ParB (Fig. 3F), suggesting that *B. subtilis* ParB can efficiently exchange CDP for CTP without dissociating from DNA as previously reported for *Caulobacter crescentus* ParB (*35*). We determined a CTP concentration for half-maximal ParB retention of about 5 to 10 μM, which is in agreement with the CTP affinity measured by ITC (Fig. 3F).

CTPγS promotes slow but robust N-gate closure even in the absence of *parS* DNA (*37*). CTP is unable to do so in wild-type ParB protein, presumably due to its hydrolysis to CDP with ParB^Intra^ being the most populated CDP state (and apo state). As expected, we found that the EQ mutants supported N-gate closure equally well with CTP and CTPγS (fig. S4), thus providing further support for the notion that the EQ mutants are defective in CTP hydrolysis and that CTP hydrolysis counteracts N-gate closure. Notably, the N-gate closure reaction was somewhat slower in E78Q and more so in E111Q when compared to wild type, possibly implying that the mutant proteins are either slightly more strongly autoinhibited (more stable ParB^Intra^) or less stably closed (less stable ParB^Inter^) (fig. S4). Together, we conclude that the EQ mutants are defective in CTP hydrolysis but support all other biochemical functions normally or near normally in vitro.

### CTP hydrolysis is dispensable for SMC recruitment but not for ParABS function

**Fig. 4.**
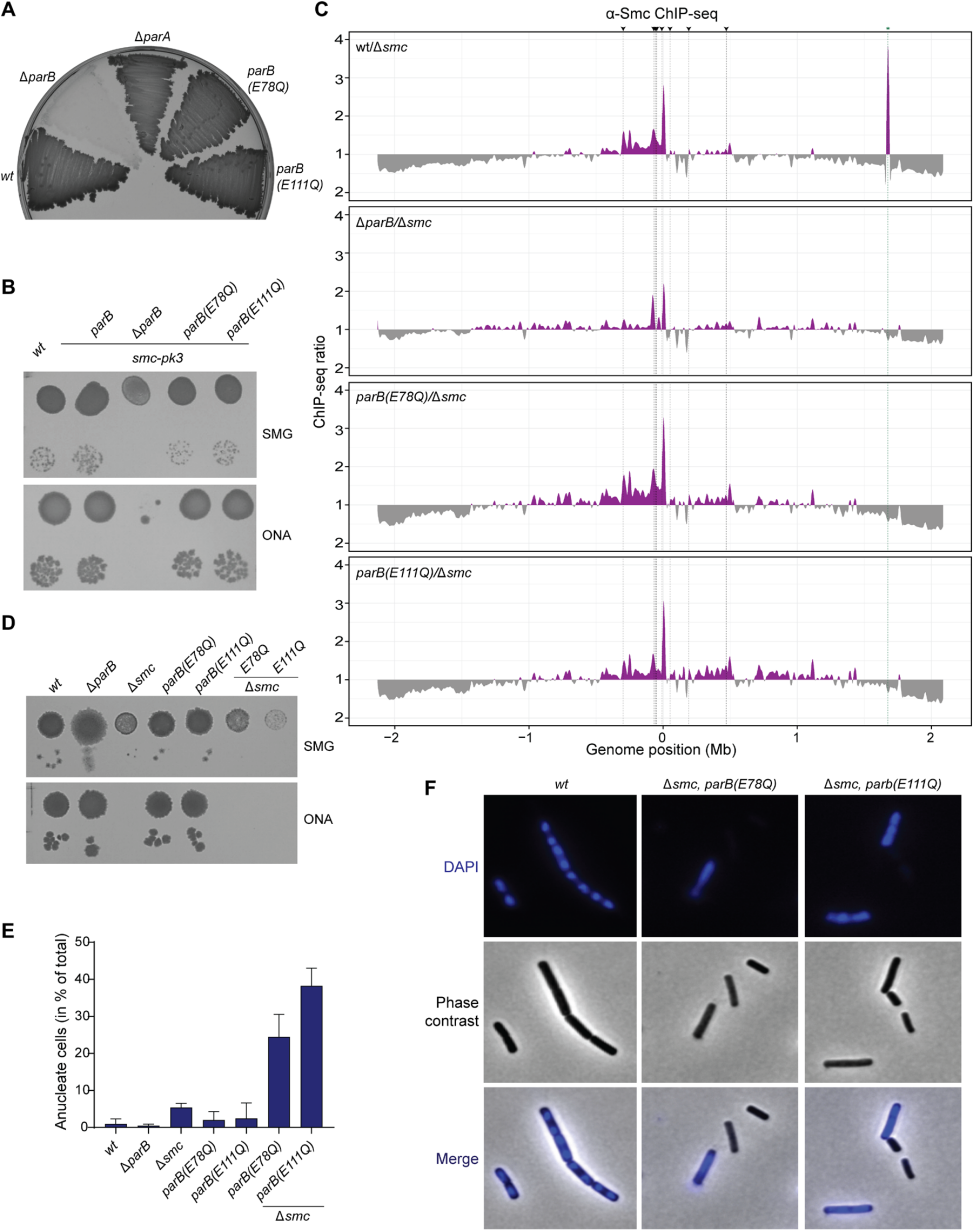
Cellular phenotypes of ParB CTP hydrolysis mutants. (**A**) Sporulation of strains with indicated *parB* alleles. (**B**) Growth assay by dilution spotting of *B. subtilis* strains carrying *parB* alleles in the hypomorphic *smc-pk3* background. (**C**) ChIP coupled to deep sequencing (ChIP-seq) using α-Smc serum. Panels show ratio plots for read counts of a given sample against that of Δ*smc* (purple color). If the ratio was below 1, then the inverse ratio was calculated and plotted inversely (gray color). Black dashed lines and arrowheads represent the eight prominent *parS* sites. Notably, the green dashed line represents enrichment of the *smc* gene presumed to be a contamination. (**D**) Growth assay of *B. subtilis* strains carrying *parB* alleles in a *smc* deletion background (“Δ*smc*”). (**E**) Quantification of the fraction of cells lacking 4′,6-diamidino-2-phenylindole (DAPI) staining (“anucleate cells”) using the ImageJ plugin microbeJ. Mean values (in percent) obtained from five fields of view with SD are reported. (**F**) Example images showing DAPI DNA staining (in blue color).

To elucidate the physiological consequences of defective CTP hydrolysis, we transferred the ParB(EQ) mutations into *B. subtilis* by allelic replacement. We observed that neither the E78Q nor the E111Q mutation resulted in a noticeable sporulation defect as judged after extended periods of incubation on nutrient-rich agar plates (Fig. 4A), implying that the regulation of DNA replication is only mildly or not at all perturbed in the mutants in contrast to the *parB* in-frame deletion mutant (Δ*parB*). Next, we combined the EQ mutations with a hypomorphic allele of the *smc* gene, *smc-pk3*, to sensitize cells for defects in chromosome organization and segregation (*24*). Again, unlike the Δ*parB* mutant, the EQ mutants supported robust growth of the *smc-pk3* strain (Fig. 4B), demonstrating that the *parB(EQ)* mutants supported chromosome segregation well, presumably by promoting the loading of Smc-ScpAB onto the chromosome. To test this more directly, we next performed ChIP using antiserum raised against the *B. subtilis* Smc protein. ChIP-seq showed that the chromosomal distribution of the Smc protein was similar in the EQ mutants and wild type and distinct from the Δ*parB* mutant (Fig. 4C). ChIP–quantitative polymerase chain reaction (qPCR) analysis of the same samples suggested that the levels of enrichment of the Smc protein were slightly reduced at the *parS-359* site and the replication origin (“*dnaA*”) in the EQ mutants, but clearly not as much reduced as in Δ*parB*, together providing further support for the notion that CTP hydrolysis is largely dispensable for Smc recruitment and loading (fig. S6B).

Next, we investigated the role of ParB CTP hydrolysis in the core function of the ParABS system. To completely rule out effects of ParB-Smc interactions on chromosome organization and segregation, we constructed the *parB(EQ)* mutants in a Δ*smc* strain. We note that the isolation of these double mutant strains was difficult. The resulting strains failed to grow on nutrient-rich medium and displayed very poor growth characteristics under nutrient limiting conditions and at reduced temperatures, thus being markedly sicker than the Δ*smc* strain (Fig. 4D). Further investigation revealed that the two double mutants [*parB(EQ)* and Δ*smc*] showed strong chromosome segregation defects with around 25 and 35% of cells lacking DNA staining (“anucleate”) in E78Q and E111Q, respectively (Fig. 4, E and F). We conclude that ParB CTP hydrolysis becomes critical for efficient chromosome segregation in the absence of Smc function in *B. subtilis*, strongly suggesting that proper function of ParABS in chromosome segregation critically relies on ParB CTP hydrolysis. Notably, we were not able to isolate Δ*smc*, Δ*parB* double mutants under the same conditions, possibly implying that ParB(EQ) proteins retain residual function even in the absence of Smc (*48*).

### CTP hydrolysis prevents excessive ParB spreading and off-target accumulation of closed ParB

**Fig. 5.**
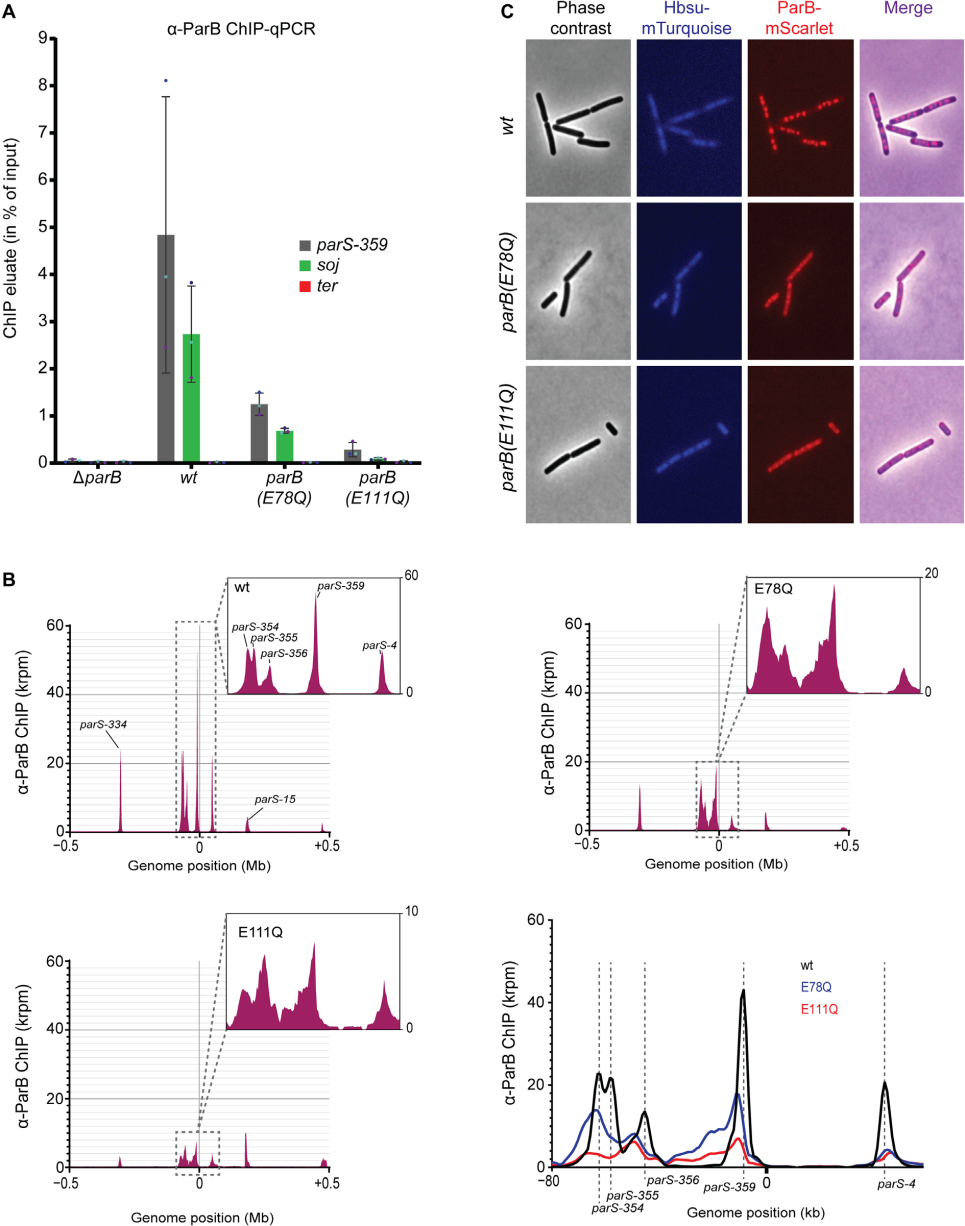
Localization defects of CTP hydrolysis–defective ParB mutants. (**A**) ChIP-qPCR using α-ParB serum. Enrichment was tested at three loci: *parS-359* (*parB*), *soj* (*parA*), and the terminus (*yocGH*). Mean values and SD from three biological replicates are reported. Individual data points are shown as dots. (**B**) ChIP-seq using α-ParB serum. Top left shows the sequence read distribution profile for wild-type ParB in a region surrounding the replication origin, and top right and bottom left show the equivalent profiles for *parB(E78Q)* and *parB(E111Q)*, respectively. Note the different scales of the y axis in the zoomed-in windows. Bottom right shows an overlay of the three profiles in a region including the *parS-354* and *parS-4* sites. Curves in the later plot have been smoothed for presentation. (**C**) Fluorescence microscopy of *B. subtilis* with different alleles of *mScarlet*-tagged *parB* (in red color) in combination with *mTurquoise*-tagged *hbs* (in blue color).

To reveal how CTP hydrolysis may promote ParABS function while being apparently dispensable for Smc recruitment, we studied the cellular localization and chromosomal distribution of ParB protein in *B. subtilis*. We performed ChIP-qPCR using antiserum raised against full-length *B. subtilis* ParB protein. The enrichment of ParB(E78Q) was clearly reduced at the *parS-359* site and the neighboring gene *parA* (*soj*) (Fig. 5A). This reduction was even more pronounced for ParB(E111Q) (Fig. 5A). Deep sequencing of the ChIP eluates resulted in reduced read counts in the EQ mutants at *parS-359* and at other *parS* sites, again with the E111Q variant displaying a more marked phenotype. Moreover, the shape of the ParB distribution at *parS* sites was clearly altered in both EQ mutants. The enriched region was significantly expanded, curiously by extending further onto one side of *parS*—away from the replication origin—but not onto the other (Fig. 5B). The width of the peak at *parS-359* increased from approximately 15 kb for wild type to roughly 40 kb for the two EQ mutants. The peak position was also slightly shifted away from the *parS* site in the direction of the extended shoulder. This suggested that the ParB(EQ) mutants exhibited excessive spreading, presumably by an increased chromosome residence time in the absence of CTP hydrolysis. This excessive spreading of ParB(EQ) was highly asymmetric, putatively because of the impediment of spreading in one direction by other chromosomal processes such as head-on transcription or DNA replication. If so, then CTP hydrolysis might reduce the frequency or impact of these encounters in wild-type cells. How this might be accomplished is unclear.

We then investigated the cellular localization of wild-type and mutant ParB-mScarlet fusion proteins by live-cell imaging (Fig. 5C). These strains also expressed a *mTurquoise*-tagged ectopic copy of *hbs*, encoding for an abundant nucleoid-associated protein with sequence-unspecific DNA binding properties. Wild-type ParB-mScarlet formed the characteristically bright foci that are known to colocalize with *parS* sites. In contrast, the two hydrolysis mutants displayed more diffusive signal with only faint foci being detected. Pretreatment with chloramphenicol (to increase the cytoplasmic space) showed that an increased fraction of the EQ variants accumulated in the cytoplasm (fig. S8A). Notably, the mScarlet fusion mildly reduced the enrichment of ParB at two tested loci as judged by ChIP-qPCR (fig. S7B).

In summary, we observed defects in the accumulation and the distribution of ParB(EQ) proteins near *parS* sites. The total level of protein in ParB-mScarlet foci decreased in the two EQ mutants, mildly in E78Q and strongly in E111Q. The occupancy of the *parS*-proximal regions was also reduced, while the occupancy of more distal positions on one of the two *parS*-flanking regions increased.

### CTP hydrolysis–defective ParB mutants accumulate in an alternate state on the chromosome

**Fig. 6.**
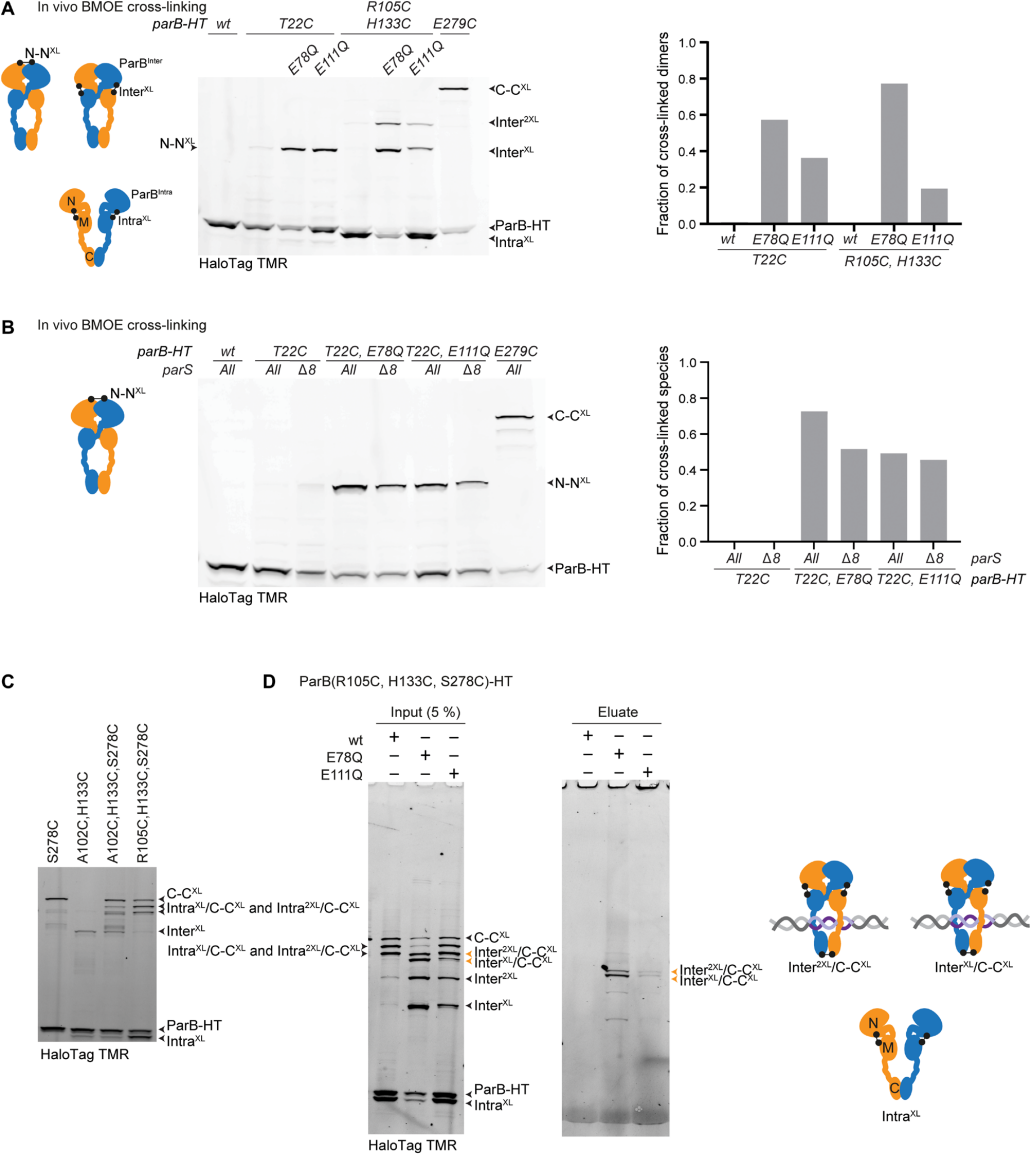
Structural states of wild-type ParB and ParB(EQ) in vivo. (**A**) In vivo BMOE cysteine cross-linking assay with ParB-HT variants. As in Fig. 1A, dimer cross-linking efficiencies were calculated by quantifying band intensities of monomeric species (ParB-HT for T22C; ParB-HT plus Intra^XL^ for R105C/H133C) and cross-linked dimeric species (N-N^XL^ for T22C; Inter^XL^ plus Inter^2XL^ for R105C/H133C) (right). Residue E279C was used a positive control for cross-linking. (**B**) Cysteine cross-linking in strains lacking *parS* sites. As in (A) using strains lacking the 8 *parS* sites. (**C**) Cysteine cross-linking in strains with multiple cysteine pairs in ParB-HT. As in (A) for identification of cross-linked species used in (D). Note that species with single and double Intra^XL^ and C-C^XL^ cannot be unambiguously identified. Bands corresponding to circular species [as seen in (D)] are not clearly discernible in the absence of hydrolysis mutations. (**D**) Chromosome entrapment assay using strains harboring ParB(R105C, H133C, S278C)–HT in combination with E78Q or E111Q. Input and output samples were analyzed by SDS-PAGE and in-gel fluorescence detection. With E78Q and E111Q, differing levels of cross-linked ParB species were found in the eluate samples. Covalent circular species of ParB-HT are denoted by arrowheads in orange colors. Notably, CTP hydrolysis–proficient ParB did not generate covalent circular species in the input samples [see also (C)]. Cross-link reversal leads to the generation of minor amounts of noncircular species in the eluates (*50*–*52*).

To elucidate the state of ParB(EQ) proteins in the cell, we next used in vivo cysteine cross-linking using ParB-HT fusion proteins. Presumably because of CTP hydrolysis dominating over N-gate closure in vivo, T22C cross-linking of otherwise wild-type ParB-HT protein was barely detectable (Figs. 1A and 6A), as reported previously (*37*). Consistently, cross-linking of R105C/H133C showed mostly the ParB^Intra^ configuration (Figs. 1A and 6A). The EQ mutants, however, robustly generated ParB N-N cross-links with T22C and ParB^Inter^ N-M cross-links with R105C/H133C (Fig. 6A). Thus, in contrast to wild-type ParB, the EQ mutants accumulated predominantly in the closed form in vivo. This finding provided further support for the notion of defective CTP hydrolysis in the EQ proteins.

The fact that EQ proteins showed more robust N-gate closure compared to wild type (Fig. 6A) implies that a large fraction of wild-type ParB in partition complexes harbors an opened N-gate. Moreover, the poor localization of the EQ mutants (and in particular of E111Q) to partition complexes indicated that they may have undergone N-gate closure without *parS* stimulation or have dissociated from *parS* DNA after N-gate closure. To discriminate between these two possibilities, we have repeated the T22C cross-linking experiment in a strain lacking eight strong *parS* sites (*49*). We found that T22C cross-linking was only mildly reduced in both EQ proteins (Fig. 6B), suggesting that a significant fraction of closed ParB clamps are formed without *parS* stimulation. CTP hydrolysis likely recovers these futile clamps in wild-type ParB.

**Fig. 7.**
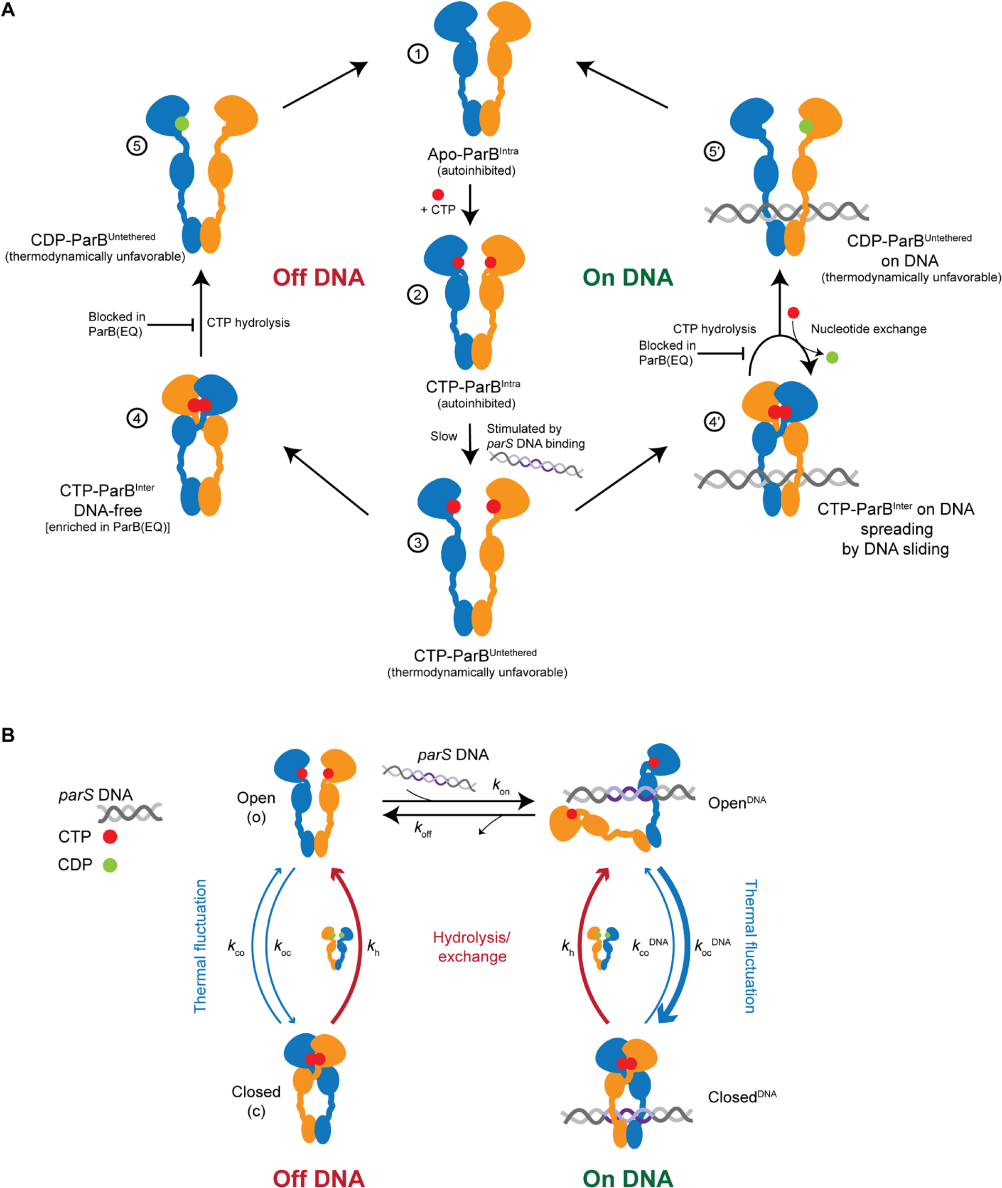
Model for partition complex assembly. (**A**) A model for ParB CTP hydrolysis and *parS* DNA catalysis in partition complex assembly. CTP binding by ParB^Intra^ (1) and (2). N and M domains spontaneously dissociate to generate ParB^Untethered^ (3). This state is thermodynamically unfavorable but stabilized by *parS* DNA binding (for details, see Fig. 2B). Subsequent clamp closure is rapid off DNA (left side) (4) and on *parS* DNA (right side) (4′). A steric clash between *parS* DNA and ParB (Fig. 2B) releases the physical contact and transfers DNA into a lumen surrounded by M and C domains (4′). ParB spreads away from *parS* DNA by one-dimensional diffusion. CTP hydrolysis leads to nucleotide exchange with or without unloading from DNA (5′). After unloading, reengagement of the N and M domains within a given ParB protomer regenerates the autoinhibited open ParB dimer (1). The partially saturated nucleotide-binding sites (5 and 5′) indicate that nucleotide release and exchange may happen before or after transition to 5 and 5′. The absence of CTP hydrolysis in ParB(EQ) mutants leads to the excessive ParB spreading by long-term chromosome residence (4′) and to the accumulation of DNA-free ParB clamps (4). (**B**) A simplified reaction scheme for CTP hydrolysis promoted enrichment of ParB at *parS* sites. Under the assumption of fast nucleotide exchange, only the CTP-bound states are considered. DNA binding and unbinding from the closed form are expected to be slow and neglected in the simple model.

As a measure for chromosome association irrespective of chromosome localization, we lastly performed chromosome entrapment assays. Briefly, we used cysteine cross-linking to generate covalently closed circular species of ParB-HT in vivo (Fig. 6C) to then detect their coisolation with chromosomal DNA in agarose beads under protein denaturing conditions (*50*, *51*). Only cross-linked species that entrap the chromosomal DNA double helix are retained in the agarose matrix. Using R105C/H133C at the N-M interface in combination with S278C at the C-C interface, we found that significant levels of cross-linked ParB(E78Q) species were retained in agarose beads (Fig. 6D). The retention of ParB(E111Q) species was comparatively low. We conclude that a considerable fraction of ParB(E78Q) entraps chromosomal DNA as a CTP-closed clamp, presumably partly near *parS* and partly distal from *parS*. Most of the ParB(E111Q) [and some of ParB(E78Q)], however, appeared to form closed ParB dimers without entrapping chromosomal DNA. Notably, as wild-type equivalents do not show robust ParB^Inter^ cross-linking with R105C/H133C, no information about hydrolysis-proficient ParB was obtained in this experiment. Together, our results indicate that ParB and ParB(EQ) occupy distinct states in the cell and when loaded onto the chromosome, which may explain at least, in part, the defects in ParABS function in the EQ mutants. A model figure summarizes these findings (Fig. 7A).

### CTP hydrolysis promotes ParB/*parS* ultra-affinity

The detailed model (Fig. 7A) could be further coarse-grained to a simple scheme for *parS* DNA association (Fig. 7B) by means of a few reasonable approximations. Only the open form of ParB can engage the substrate, whereas closed ParB cannot bind or release DNA substrates, because steric clashes make these processes extremely unlikely, if not impossible. Furthermore, ParB without nucleotides is a transient, poorly populated state, as could be inferred from the longer persistence of already bound ParB on DNA in the presence of CTP in BLI experiments (Fig. 3F). Thus, we captured the transition from CDP-bound to CTP-bound ParB by means of a simple transition. The resulting model can be analytically solved (see Materials and Methods), giving an estimate of the “apparent,” observed, dissociation constant ($K_{d}^{\text{obs}}$) of ParB for DNA

$$K_{d}^{\text{obs}}\approx K_{d}^{\text{open}}\frac{k_{h}}{k_{\text{oc}}^{\text{DNA}}+k_{h}}$$

The *parS* sequence greatly accelerates ParB closure ($k_{\text{oc}}^{\text{parS}}\gg k_{h}$), thus decreasing the value of the observed dissociation constant. In the case of nonspecific DNA, however, the rate of ParB closure is unaffected and likely slower than the hydrolysis rate, resulting in a dissociation constant that is the same as the one of open ParB. The same observed dissociation constant also applies to hydrolysis-deficient mutants at equilibrium (see Materials and Methods).

## DISCUSSION

The partition complex is critical for ParABS function. The assembly has been intensely studied over several decades. The recently found requirement for CTP binding by ParB in partition complex assembly opened new avenues for investigation. Here, we focused on the mechanistic basis of *parS* DNA catalysis of ParB clamp loading and on the physiological role of ParB CTP hydrolysis in partition complex assembly and chromosome segregation.

### *parS* DNA: A B-form DNA double helix as catalyst

Ribozymes are naturally occurring nucleic acid catalysts that generally use folded single-stranded RNA to catalyze a variety of chemical reactions. Unlike ribozymes, *parS* DNA appears to work as catalyst in the standard B-form of double-stranded DNA. On the basis of structural information and chemical cross-linking, we here propose a molecular mechanism for *parS* catalysis. We found that ParB dimers are unable to engage with both halves of the palindromic *parS* site when adopting the predominant conformation with tethered N and M domains, ParB^Intra^ and ParB^Inter^. Partial unfolding of ParB appears to be needed for full engagement of *parS* DNA. This idea explains how *parS* DNA selectively stabilizes an intermediate of the reaction, while also binding to the reactant of the reaction, albeit with reduced affinity. Available cocrystal structures are consistent with the notion of a steric clash between *parS*-bound ParB chains. The structure of *Helicobacter pylori* ParB (lacking the C domain) bound to *parS* (PDB: 4UMK) (*38*) shows partially unfolded N domains (in four variations). Similarly, a C-terminally truncated variant of *C. crescentus* ParB (PDB: 6T1F) displays partial unfolding in one of the two *parS*-bound chains. These structures imply that *parS* DNA binding sufficiently compensates for the energetic investment in the partial unfolding of the N domain. The fact that a cysteine pair at the N-M interface (A102C/L134C) hinders sporulation and like another cysteine pair (A102C/H133C) leads to elevated levels of the ParB^Inter^ conformation (fig. S1C) supports the notion that this interface is indeed important for function and conformational control. We also demonstrated that the apposition of *parS* half sites is critical for catalysis. Spacer addition not only eliminated catalysis but also led to inhibition of the reaction, possibly due to improved binding of ParB^Intra^ dimers to the modified *parS* sites (Fig. 2B). This paradigm puts forward a strategy for engineering self-loading clamps with altered targeting specificity by repurposing sequence-specific or sequence-guided DNA binding proteins, eventually allowing for flexible chromosome labeling and DNA detection for diagnostic purposes.

### ParB CTP hydrolysis in partition complex assembly

We showed here that CTP hydrolysis by ParB is dispensable for some functions of ParABS in *B. subtilis* and important for others. Sporulation and Smc recruitment were largely unperturbed in the CTP hydrolysis–deficient mutants E78Q and E111Q (Fig. 4). In contrast, ParB mutants interfering with CTP binding (G77S and R80A) are deficient in Smc recruitment (*37*, *52*) and sporulation (*28*, *42*). The robustness of Smc recruitment is remarkable, particularly when considering the mediocre enrichment of ParB(E111Q) at *parS* sites. Conceivably, the low levels of ParB(EQ) are compensated by it being trapped in a state that efficiently supports Smc recruitment. It is thus tempting to speculate that the CTP-ParB^Inter^ state is proficient in Smc loading and conceivably even solely responsible for it. In case of sporulation, any closed ParB clamps accumulating off the chromosome may also contribute to the conversion of ParA ATP dimers to monomers, which is a prerequisite for sporulation, since a HTH mutant appears to support sporulation quite well (*24*). In contrast, chromosome segregation in the absence of *smc* was strongly compromised in either CTP hydrolysis mutant, indicating defective ParABS function (Fig. 4). This defect likely explains why the CTPase activity is highly conserved and maintained over extended periods of time during evolution.

Explanations for the poor function of ParABS in the absence of CTP hydrolysis include (i) an inadequate assembly of the partition complex and (ii) the accumulation of ParB in a state that is incompatible with proper ParABS function. We present evidence for either scenario. The notion of altered states of ParB in the EQ mutants is supported by our cysteine cross-linking (Fig. 6): While CTP hydrolysis–proficient wild-type ParB shows only low levels of ParB^Inter^ clamp formation in vivo (Figs. 1A and 6A), the EQ mutant variants predominantly adopt this state. It is also consistent with a recent report of CTP-controlled stimulation of ATP hydrolysis by ParA in the F plasmid ParABS system (*53*). Future work will have to establish, which form of ParB supports Smc loading and which one promotes ParA ATP hydrolysis. This may potentially establish temporal or spatial pattern of chromosome organization, segregation, and DNA replication.

Several observations point to defects in concentrating ParB in partition complexes in E78Q and E111Q. ChIP and fluorescence imaging revealed a reduced enrichment and broadened distribution of ParB(EQ) proteins at and near *parS* sites. CTP hydrolysis conceivably supports partition complex assembly by recycling ParB^Inter^ species. We envision two pools of ParB^Inter^ that may require recycling: ParB clamps that have excessively spread away from the *parS* loading site and clamps that have undergone N-gate closure without *parS* DNA stimulation, either becoming locked off the chromosome or trapped on the chromosome at large distances from *parS* sites (Fig. 7A). These clamps would be permanently lost from the pool of productive ParB protein without the ability to hydrolyze CTP. *parS*-independent clamp closure readily occurs in vitro at least with *B. subtilis* ParB as indicated by the low but appreciable rates of CTP hydrolysis without *parS* DNA stimulation and by the slow but robust closure of the N-gate with the help of CTPγS in the absence of *parS* DNA. Similarly, we detected closed ParB(E78Q) clamps in cells lacking *parS* sites (Fig. 6B). Nevertheless, we cannot exclude the possibility that the poor enrichment at *parS* sites is caused at least, in part, by indirect consequences of the E78Q and E111Q mutations on ParB activity, for example, by reducing N-gate stability or ParB autoinhibition.

We also described ParB behavior by a simple reaction scheme (Fig. 7B). Because of CTP hydrolysis, ParB exhibits an enhanced affinity for *parS* sequences, beyond the one that would be possible at equilibrium (e.g., by hydrolysis-deficient mutants). This ultra-affinity (*54*) corresponds to a roughly 50-fold reduction of the dissociation constant, from an estimate of the hydrolysis and *parS*-catalyzed ParB closure rates [*k*_h_ ~1/min; $k_{\text{oc}}^{\text{parS}}$ ~1/s; (*37*)]. The enrichment of ParB on the *parS* flanking regions is a consequence of this effect because the increased amount of ParB bound to *parS* can subsequently diffuse away, thus populating the nearby nonspecific DNA sequences.

Wild-type ParB barely adopts the clamp state in vivo despite being mostly localized in ParB foci (*9*, *55*, *56*). Mechanisms other than DNA entrapment may thus contribute to the retention of wild-type ParB protein in the partition complex, such as contacts between (open) ParB dimers (*3*, *28*, *30*, *57*). DNA unloading upon CTP hydrolysis is prevented in vitro by the presence of CTP in case of *B. subtilis* ParB (Fig. 3F) and *C. crescentus* ParB (*35*). This implies that CDP is readily substituted for CTP without ParB unloading from the chromosome (Fig. 7B). It will be interesting to establish whether this nucleotide exchange reaction requires localization of ParB to the partition complex and DNA entrapment by ParB. If so, then this feature would selectively eliminate off-target clamps or DNA-free clamps, respectively, thus further enhancing *parS*-proximal enrichment of ParB.

We conclude that *parS* DNA catalysis and ParB CTP hydrolysis lead to ParB/*parS* ultra-affinity and superconcentrate ParB within the partition complex. The unusually high concentration of ParB (*58*) is likely required to optimally support chromosome and plasmid segregation by the ParABS diffusion-ratchet mechanism but might not be essential for other cellular functions of ParABS such as Smc recruitment.

## MATERIALS AND METHODS

### *B. subtilis* strains and growth

The *B. subtilis* 1A700 isolate was used for all experiments. *B. subtilis* was transformed via natural competence and homologous recombination as described in (*59*) and grown on minimal medium (SMG) agar plates with appropriate antibiotic selection. Transformants were next checked by PCR and Sanger sequencing. Tagging of ParB (with HT or mScarlet) or Hbsu (with mTurquoise) proteins was performed at the C terminus. Genotypes of strains used in this study are listed in table S1.

For spotting assays, the cells were cultured in SMG medium at 37°C to stationary phase and 9^2^- and 9^5^-fold dilutions were spotted onto ONA (Oxoid nutrient agar) (~16-hour incubation) and SMG (~24-hour incubation) agar plates.

### Expression and purification full-length *B. subtilis* ParB protein

Expression constructs were prepared in pET-28 derived plasmids by Golden-Gate cloning. Untagged recombinant proteins were produced in *Escherichia coli BL21-Gold (DE3)* grown in ZYM-5052 autoinduction media at 24°C for 24 hours. Purification of full-length ParB protein was performed as described before in (*37*). Briefly, cells were lysed by sonication in buffer A [1 mM EDTA (pH 8), 500 mM NaCl, 50 mM tris-HCl (pH 7.5), 5 mM β-mercaptoethanol, 5% (v/v) glycerol, and protease inhibitor cocktail (PIC; Sigma-Aldrich)]. Ammonium sulfate was added to the supernatant to 40% (w/v) saturation and kept at 4°C for 30 min while stirring. The sample was then centrifuged, the supernatant was collected, and ammonium sulfate was added to 50% (w/v) saturation and kept at 4°C for 30 min while stirring. The pellet was then collected by centrifugation and dissolved in buffer B [50 mM tris-HCl (pH 7.5), 1 mM EDTA (pH 8), and 2 mM β-mercaptoethanol]. The sample was additionally diluted with buffer B to a conductivity of 18 mS/cm and loaded onto a Heparin column (GE Healthcare). The protein was eluted with a linear gradient of buffer B containing 1 M NaCl. Peak fractions were collected and diluted with buffer B to a conductivity of 18 mS/cm and loaded onto HiTrap SP columns (GE Healthcare). A linear gradient of buffer B containing 1 M NaCl was used for elution. Peak fractions were collected and directly loaded onto a Superdex 200 16/600 pg column (GE Healthcare) preequilibrated in 300 mM NaCl and 50 mM tris-HCl (pH 7.5). For cysteine mutant ParB, 1 mM TCEP (tris(2-carboxyethyl)phosphine) was added to the gel filtration buffer. For ITC measurements, peak fractions were collected, diluted 1:1 with buffer containing 50 mM tris-HCl (pH 7.5) and 10 mM MgCl_2_ to bring the final buffer to 150 mM NaCl, 50 mM tris-HCl (pH 7.5), and 5 mM MgCl_2_, and directly used for measurements.

### CTPγS

CTPγS was custom-synthesized and purified by reversed-phase chromatography to a final purity of 90.6% by Jena Biosciences (Jena, Germany). A stock solution at a concentration of 100 mM (pH-adjusted to 8.0 by addition of NaOH) was aliquoted and stored at −80°C.

### Isothermal titration calorimetry

The measurement was performed using MicroCal iTC200 (GE Healthcare Life Sciences). The instrument was precooled to 4°C. All measurements were made in buffer containing 150 mM NaCl, 50 mM tris-HCl (pH 7.5), and 5 mM MgCl_2_. Both measurement cell and injection syringe were subjected to a series of washes with buffer. Two hundred eighty microliters of the protein solution at 80 to 120 μM monomer concentration was added to the measurement cell, and the injection syringe was filled with buffer containing 2 mM CTP or buffer only. Measurements were taken with an initial delay of 180 s, and the settings of the instrument were adjusted to reference power of 5 μcal/s, a stirring velocity of 1000 rpm, and a “high feedback” mode. Raw data were integrated to kilocalories per mole, presented as a Wiseman plot, and wherever possible, regression curves were calculated according to a 1:1 nucleotide to ParB monomer binding model. Origin (GE Healthcare) was used for fitting results from the measurements by using the following equation

$$∆Q(i)=Q(i)+\frac{{\mathrm{dV}}_{i}}{{\mathrm{dV}}_{o}}[\frac{Q_{i}+Q(i-1)}{2}]-Q(i-1)$$

*V_i_* is the injection volume of ligand (nucleotides), *V_o_* is the volume of the cell, *Q*(*i*) is the heat released from the *i*th injection that is, in turn, calculated using the following equation

$$Q=\frac{M_{t}∆HV_{o}}{2}[1+\frac{X_{t}}{M_{t}}\frac{1}{KM_{t}}-\sqrt{{\left(1+\frac{X_{t}}{M_{t}}+\frac{1}{KM_{t}}\right)}^{2}-\frac{4X_{t}}{M_{t}}}]$$

*K* is the binding constant, Δ*H* is the molar heat of ligand binding, *X_t_* is bulk concentration of nucleotide, and *M_t_* is the bulk concentration of ParB (moles per liter) in *V_o_*. *K* and Δ*H* were estimated by Origin, and Δ*Q*(*i*) for each injection was calculated and compared to the measured heat. *K* and Δ*H* estimates were then improved using standard Marquardt methods. Several iterations were performed until the fit could no longer be improved.

### Measurement of CTP hydrolysis by malachite green colorimetric detection

Mixtures of CTP (2×) with or without *^parS^*DNA_40_ (2×) in reaction buffer [150 mM NaCl, 50 mM tris (pH 7.5), and 5 mM MgCl_2_] were prepared on ice. Protein solutions (2×) in reaction buffer were also prepared on ice. CTP/*^parS^*DNA_40_ premix (10 μl) was added to protein solution (10 μl) using BenchSmart 96 (Rainin) dispenser robot and mixed by pipetting. After mixing, samples (containing 1 mM CTP, 1 μM *^parS^*DNA_40_, and 10 μM protein) were placed in a PCR machine and set to incubate at 25°C for 1 hour. In parallel, phosphate blanks were prepared for each experiment. Samples were diluted fourfold by the addition of 60 μl of water, then mixed with 20 μl of working reagent (Sigma-Aldrich), and transferred to a flat-bottom 96-well plate. The plate was left to incubate for 30 min at 25°C, and the absorbance was then read at a wavelength of 620 nm. Absorbance values from the phosphate standard samples were used to plot an optical density at 620 nm (OD_620_) versus phosphate concentration standard curve. Raw values were converted to rate values using the standard curve. Absolute rates were calculated by normalizing for protein concentration. Mean values and SD were calculated from three repeat experiments. Graphs were plotted on GraphPad Prism for presentation.

### Forty-nucleotide, double-stranded *^parS^*DNA_40_ preparation

Complementary strands of oligonucleotides (at 100 μM each) (for sequences, see table S2) were mixed 1:1 and heated to 95°C for 10 min and then left to cool down to 25°C.

### In vitro cross-linking

Mixtures (2×) of nucleotide triphosphate (NTP) with or without *^parS^*DNA_40_ were prepared in reaction buffer [150 mM NaCl, 50 mM tris-HCl (pH 7.5), and 5 mM MgCl_2_] and kept at room temperature (RT) for 5 min. Protein solution [2× in 300 mM NaCl and 50 mM tris-HCl (pH 7.5)] was added to a final concentration of 10 μM, and the mixture (containing 1 mM NTP with or without 1 μM *^parS^*DNA_40_) was incubated for an additional 5 min at RT. BMOE (1 mM; from a 20 mM stock solution) was added to the samples. After 5 min at RT, samples were quenched with β-mercaptoethanol (23 mM final), loading dye was added, and samples were incubated at 70°C for 5 min and loaded onto bis-tris 4 to 12% gradient gels (Thermo Fisher Scientific). Bands were stained by Coomassie Brilliant Blue (CBB), and relative band intensity was quantified by scanning and semiautomated analysis in ImageQuant (GE Healthcare).

### In vivo cross-linking

Culture, grown overnight in 0.5% (w/v) glucose containing LB, was used to inoculate fresh LB medium to an OD_600_ of 0.005 and were grown at 37°C to midexponential phase (OD_600_ of 0.3). Around 70 g of ice cubes were added directly to the culture flask, and the cells were harvested by centrifugation for 5 min at 14,000*g* at 4°C. The cell pellet was washed in cold PBSG [phosphate-buffered saline (PBS) with 0.1% (v/v) glycerol]. Cells were pelleted again and resuspended in 1 ml of cold PBSG. A total of 1.25 OD units were collected and resuspended in 30 μl of cold PBSG. BMOE (0.5 mM) was then added to the samples, mixed by pipetting, and placed on ice for 15 min. β-Mercaptoethanol (0.5 mM) was added, and the samples were incubated on ice for 3 min to quench the reaction. The following reagents were added to the given final concentrations: Benzonase (750 U/ml; Sigma-Aldrich), 5 μM HaloTag-TMR (Tetramethylrhodamine) ligand (Promega), Ready-Lyse Lysozyme (47 U/μl; Epicentre), and 1× PIC (Sigma-Aldrich). The cells were then lysed at 37°C in the dark for 30 min, loading dye containing 5% (v/v) β-mercaptoethanol was added, and the samples were incubated at 70°C for 5 min. Ten microliters of each sample was loaded onto a 4 to 12% bis-tris gel (Thermo Fisher Scientific). Following SDS-PAGE, gels were imaged on an Amersham Typhoon (GE Healthcare) with Cy3 DIGE filter setup. Bands were quantified using ImageQuant (GE Healthcare).

### Chromosome entrapment assay

The assay was performed as described in (*50*, *51*) using agarose beads. Briefly, cells were grown and harvested, and 3.75 OD units was cross-linked as described above (in vivo cross-linking). The sample was split into two aliquots (2:3 for the output/beads and 1:3 for input). To each input sample, 5 μl of Mastermix-1 was added. Mastermix-1 contains (per one input sample) 400 U of Ready-Lyse Lysozyme, 12.5 U of Benzonase, 1 μM HaloTag-TMR ligand, 0.5 μl of PIC, and 0.9 μl of 1× PBS. The input samples are then incubated for 25 min at 37°C covered from light, an equal volume of 2× protein loading dye was added, and the samples were stored at −20°C.

For agarose bead preparation, 1 μl of PIC and 9 μl of Dynabeads M-280 Streptavidin (Thermo Fisher Scientific) were added to each output (“beads”) sample. One hundred microliters of preheated 2% low melt agarose was then added and immediately followed by 700 μl of mineral oil (preheated to 4°C). The sample was then vortexed vigorously for 2 min on ice. Each sample was treated quickly and separately.

The output (beads) samples were then spun down at 10,000*g* at RT, and the supernatant (oil) was removed as much as possible. The beads were then resuspended in 1 ml of PBSG (0.1% glycerol) very gently to prevent the release of cells from the agarose beads.

To each output sample, Ready-Lyse Lysozyme (4 U/μl), 1 μM HaloTag-TMR ligand, 5 μl of PIC, and 1 mM EDTA were added (same as Mastermix-1 but without Benzonase). The output samples are then incubated for 25 min at 37°C covered from light. Next, the beads were washed twice with 1 ml of RT PBSG and with TES buffer [50 mM tris-HCL (pH 7.5), 10 mM EDTA, and 1% SDS] three times, with the first time incubated for 1 hour at RT with moderate shaking (500 rpm), and the following two incubations were performed for 30 min. The beads were resuspended in 1 ml of TES and incubated overnight at 4°C on a rotating wheel and covered from light.

The next day, the beads were washed twice with 1× PBS at RT to remove EDTA and SDS and resuspended in 100 μl of PBS. One microliter of SmDnase (750 U) was added to each output sample and incubated for 1 hour at 37°C with moderate shaking and covered from light. The samples were next incubated for 1 min at 70°C at 14,000 rpm shaking to melt the agarose plugs and then transferred onto ice and incubated for 5 min. The samples were spun down at maximum speed for 15 min at 4°C, and the supernatant was transferred to an acetate spin column and spun down for additional 5 min at RT at maximum speed to recover as much liquid as possible. The approximate recovered volume is 100 μl that was then diluted to 1 ml with water and 10 μl of 2% sodium deoxycholate, and BSA (3.3 mg/ml) was added and incubated for 30 min at 4°C protected from light. After incubation, 120 μl of 80% trichloroacetic acid solution was added and incubated for 2 hours on ice and covered from light. The samples are then spun down for 15 min at maximum speed at 4°C. The precipitate was collected and resuspended in 10 μl of 1× loading dye. The input samples were thawed and boiled with the output samples for 5 min at 95°C. Five percent of the input and all of the output volume were loaded on an SDS-PAGE using 8 to 12% Novex Wedge Well tris-glycine gel (Thermo Fisher Scientific). The gel was then imaged on Amersham Typhoon (GE Healthcare) with Cy3 filter.

### Biolayer interferometry

Measurements were performed in a buffer containing 150 mM NaCl, 50 mM tris-HCl (pH 7.5), and 5 mM MgCl_2_ on BLItz machine (FortéBio Sartorius). Streptavidin-coated biosensors were used in all the measurements and were hydrated in the reaction buffer for 10 min before loading. A baseline was first recorded by equilibrating the biosensor in 250 μl of reaction buffer in a black 0.5-ml Eppendorf tube for 30 s. Four microliters of 100 nM biotin-labeled double-stranded *parS* or mut-*parS* DNA_169bp_ was then loaded on the biosensor for 5 min. After the DNA loading phase, the biosensor was washed once with the reaction buffer and once with the reaction buffer containing 1 mM NTP. Next, 2× ParB solution and 2× NTP solution were mixed 1:1 (final concentration of 1 μM parB and 1 mM NTP), and 4 μl of the mixture was loaded immediately on the biosensor for 2 min. The dissociation phase is then carried for 5 min in 250 μl of protein-free reaction buffer with or without NTP. All measurements were analyzed on the BLItz analysis software and replotted on GraphPad Prism for presentation.

### Chromatin immunoprecipitation

ChIP samples were prepared as described previously (*60*) with minor modifications. Cells were grown in 200 ml of minimal media (SMG) at 37°C until midexponential phase (OD_600_ = 0.03). Next, 20 ml of fixation buffer F was added [50 mM tris-HCl (pH 7.4), 100 mM NaCl, 0.5 mM EGTA (pH 8.0), 1 mM EDTA (pH 8.0), and 10% (w/v) formaldehyde] for 30 min at RT with occasional shaking. Cells were harvested by filtration and washed in 1× PBS. Each sample was adjusted for 2 OD_600_ units (2 ml at OD_600_ = 1) and resuspended in TSEMS lysis buffer [50 mM tris (pH 7.4), 50 mM NaCl, 10 mM EDTA (pH 8.0), 0.5 M sucrose and PIC (Sigma-Aldrich), and lysozyme (6 mg/ml) from chicken egg white (Sigma-Aldrich)]. After 30 min of incubation at 37°C with vigorous shaking, protoplasts were washed again in 2 ml of TSEMS, resuspended in 1 ml of TSEMS, split into three aliquots, pelleted, flash-frozen, and stored at −80°C for further use.

For ChIP-qPCR, each pellet was resuspended in 2 ml of buffer L [50 mM Hepes-KOH (pH 7.5), 140 mM NaCl, 1 mM EDTA (pH 8.0), 1% (v/v) Triton X-100, 0.1% (w/v) sodium deoxycholate, ribonuclease A (0.1 mg/ml), and PIC (Sigma-Aldrich)] and transferred to 5-ml round-bottom tubes. Cells were sonicated three times for 20 s on a Bandelin SONOPULS with a MS72 tip (settings: 90% pulse and 35% power output). The lysates were next transferred into 2-ml tubes and centrifuged for 10 min at 21,000*g* at 4°C. Eight hundred microliters of the supernatant was used as input (IP), and 200 μl was kept as whole-cell extract (WCE). The WCE tubes are frozen at −20°C for later.

For the IP, antibody serum was preincubated with Protein G–coupled dynabeads (Invitrogen) in 1:1 ratio for 2 hours at 4°C on a rotating wheel. Next, the beads were washed in buffer L, and 50 μl were aliquoted to each sample tube. Samples were incubated with the beads for 2 hours at 4°C with rotation. After incubation, all samples were subjected to a series of washes with buffer L, buffer L5 (buffer L containing 500 mM NaCl), buffer W [10 mM tris-HCl (pH 8.0), 250 mM LiCl, 0.5% (v/v) NP-40, 0.5% (w/v) Na deoxycholate, and 1 mM EDTA (pH 8.0)], and buffer TE [10 mM tris-HCl (pH 8.0) and 1 mM EDTA (pH 8.0)]. After washing, the beads were resuspended in 520 μl of buffer TES [50 mM tris-HCl (pH 8.0), 10 mM EDTA (pH 8.0), and 1% (w/v) SDS]. The WCE are thawed, and 300 μl of TES and 20 μl of 10% SDS were also added. Both tubes were incubated overnight at 65°C with vigorous shaking to reverse the formaldehyde cross-linking. The next day, phenol-chloroform DNA extraction was performed to purify the decross-linked DNA. Samples were transferred to screw cap 1.5-ml tubes and mixed vigorously with 500 μl of phenol equilibrated with buffer [10 mM tris-HCl (pH 8.0) and 1 mM EDTA]. After centrifugation (10 min at RT and 13,000 rpm), 450 μl of the aqueous phase was transferred to a new screw cap tube and mixed with equal volume of chloroform, followed by centrifugation. Four hundred microliters of aqueous phase was then recovered for DNA precipitation with 1 ml of 100% ethanol (2.5× volume), 40 μl of 3 M NaOAc (0.1× volume), and 1.2 μl of GlycoBlue and incubated for 20 min at −20°C. Last, samples were centrifuged for 10 min at 20,000*g* at RT, and the pellets were purified with a PCR purification kit, eluting in 50 μl of buffer EB.

For qPCR, 1:10 and 1:1000 dilutions in water of IP and WCE were prepared, respectively. Each 10 μl of reaction was prepared in duplicate (5 μl of Takyon SYBR MasterMix, 1 μl of 3 μM primer pair, and 4 μl of DNA) and run in Rotor-Gene Q machine (QIAGEN). Primer sequences are listed in the table S2. Raw data were analyzed using PCR Miner server (http://ewindup.info). For IP samples for ChIP-seq, the procedure was the same as for ChIP-qPCR, except for resuspending the pellets in 1 ml of buffer L and sonication in a Covaris E220 water bath sonicator for 5 min at 4°C, 100 W, 200 cycles, 10% load, and water level 5.

For deep sequencing, the DNA libraries were prepared by the Genomic Technologies Facility (GTF) at the University of Lausanne. Briefly, the DNA was fragmented by sonication (Covaris S2) to fragment sizes ranging from 220 to 250 bp. DNA libraries were prepared using the Ovation Ultralow Library Systems V2 Kit (NuGEN) including 15 cycles of PCR amplification. Eighty to one hundred million single-end sequence reads were obtained on a HiSeq 4000 (Illumina) with 151-bp read length.

### Processing of ChIP-seq reads

Reads were mapped to *B. subtilis* genome NC_000964.3 with bowtie2 using the default mode. Subsequent data analysis was performed using SeqMonk (www.bioinformatics.babraham.ac.uk/projects/seqmonk/). A bin size of 1 kb was used.

### Western blot

Cells were grown and harvested following the same protocol for in vivo cross-linking (see above). A total of 1.25 OD units were collected and resuspended in 45 μl of cold PBSG. Four hundred units of Ready-Lyse Lysozyme (Epicentre), 12.5 U of Benzonase (Sigma-Aldrich), and 0.5 μl of PIC (Sigma-Aldrich) were added and incubated for 30 min at 37°C. Next, 1× Loading Dye containing dithiothreitol (200 mM final) was added to the sample and incubated at 70°C for 10 min. The samples were run on Novex WedgeWell 4 to 12% tris-glycine (Invitrogen) gel at 200 V for 1 hour in 1× Laemmli buffer. Protein bands were then transferred onto a nitrocellulose membrane (GE Healthcare) using wet transfer. Blocking solution (5% dry nonfat milk powder in tris-buffered saline with 0.05% Tween 20) was added to the membrane and left to incubate for 1 hour. Dilution (1:10,000) of rabbit polyclonal serum against *B. subtilis* ParB was used as a primary antibody for immunoblotting and left to incubate with the membrane for 2 hours. Next, the membrane was developed by incubating with horseradish peroxidase–coupled secondary antibodies for 2 hours. Chemiluminescence reagents (Amersham ECL Western Blotting Detection Reagent) were added, and FUSION FX7 (VILBER) imaging system was used to visualize the membrane.

### Mathematical model of ParB-DNA association

The steady-state solution of the model in Fig. 7B relies on the absence of net fluxes over the reaction network. Thus, the concentration of unbound ParB in the open state is related to the concentration of unbound ParB in the closed state by the relation

$${\left[\text{ParB}\right]}_{\text{open}}=\frac{k_{\text{co}}+k_{h}}{k_{\text{oc}}}{\left[\text{ParB}\right]}_{\text{closed}}$$

The total amount of unbound ParB is

$$[\text{ParB}]={\left[\text{ParB}\right]}_{\text{open}}+{\left[\text{ParB}\right]}_{\text{closed}}$$

that can be used to write

$${\left[\text{ParB}\right]}_{\text{open}}=\frac{k_{\text{co}}+k_{h}}{k_{\text{oc}}+k_{\text{co}}+k_{h}}[\text{ParB}]$$

Similarly,

$${\left[\text{ParB}∙\text{DNA}\right]}_{\text{open}}=\frac{k_{\text{co}}^{\text{DNA}}+k_{h}}{k_{\text{oc}}^{\text{DNA}}+k_{\text{co}}^{\text{DNA}}+k_{h}}[\text{ParB}∙\text{DNA}]$$

where [*ParB* ∙ DNA] is the total concentration of ParB bound to DNA. Here, we assumed that the hydrolysis rate is not significantly affected by the bound DNA.

From these relations, it is possible to determine the observed dissociation constant of ParB (independently of its open or closed state) for DNA

$$K_{d}^{\text{obs}}=\frac{\left[\text{ParB}][\text{DNA}\right]}{\left[\text{ParB}∙\text{DNA}\right]}=K_{d}^{\text{open}}\frac{k_{\text{co}}^{\text{DNA}}+k_{h}}{k_{\text{co}}+k_{h}}\;\frac{k_{\text{oc}}+k_{\text{co}}+k_{h}}{k_{\text{oc}}^{\text{DNA}}+k_{\text{co}}^{\text{DNA}}+k_{h}}$$

There are three cases:

1) *k*_h_ ≈ 0:Hydrolysis-deficient mutant. The dissociation constant reduces to

2)

Kdobs=KdopenkcoDNAkco koc+kcokocDNA+kcoDNA=Kdopen1+kockco1+kocDNAkcoDNA

If DNA (whether *parS* of nonspecific sequences) only accelerates the transitions between the open and closed states but does not alter significantly their equilibrium, then

kocDNAkcoDNA≈kockco

and consequently

$$K_{d}^{\text{obs},\text{mut}}\approx K_{d}^{\text{open}}$$

3) Nonspecific DNA. In this case, the transitions between the open and closed states is unaffected by substrate binding

4)

$$k_{\text{oc}}^{\text{DNA}}\approx k_{\text{oc}}\;k_{\text{co}}^{\text{DNA}}\approx k_{\text{co}}$$

and again, as a consequence

$$K_{d}^{\text{obs},\text{non}-\text{specific}\;\text{DNA}}\approx K_{d}^{\text{open}}$$

5) *parS* DNA. We know that

6)

$$k_{\text{oc}}^{\text{parS}}\gg k_{\text{co}}^{\text{parS}}\;k_{\text{oc}}^{\text{parS}}\gg k_{h}$$

the first relation is due to the spontaneous closure of hydrolysis-deficient mutants (with CTP) or wild-type protein (with a nonhydrolyzable analog) (fig. S4), telling that the closed state is lower in free energy than the open state; the second obtained from the observation that ~40-fold substoichiometric amounts of *parS* DNA are sufficient to fully stimulate CTP hydrolysis and N-gate closure, meaning that a step other than closure (presumably hydrolysis) is rate-limiting (*37*). Furthermore, considering that for free ParB it is *k*_h_ ≫ *k*_co_, *k*_oc_ to ensure proper recycling, we have

$$K_{d}^{\text{obs},\text{parS}}\approx K_{d}^{\text{open}}\frac{k_{h}}{k_{\text{oc}}^{\text{parS}}+k_{h}}$$

that is much smaller than $K_{d}^{\text{open}}$.

## Notes

View/request a protocol for this paper from *Bio-protocol*.
