# Supplementary material for: Relief of ParB autoinhibition by parS DNA catalysis and recycling of ParB by CTP hydrolysis promote bacterial centromere assembly
Source: Sci Adv. 2021 Oct 6;7(41):eabj2854. doi: 10.1126/sciadv.abj2854 (PMC8494293; doi:10.1126/sciadv.abj2854)
Supplement: Supplementary file 1 — Figs. S1 to S8 Tables S1 and S2 [file sciadv.abj2854_sm.pdf]

Science Advances
AAAS

Supplementary Materials for

Relief of ParB autoinhibition by *parS* DNA catalysis and recycling of ParB by CTP hydrolysis promote bacterial centromere assembly

Hammam Antar, Young-Min Soh, Stefano Zamuner, Florian P. Bock, Anna Anchimiuk, Paolo De Los Rios, Stephan Gruber*

*Corresponding author. Email: stephan.gruber@unil.ch

Published 6 October 2021, *Sci. Adv.* **7**, eabj2854 (2021)
DOI: 10.1126/sciadv.abj2854

This PDF file includes:

Figs. S1 to S8
Tables S1 and S2

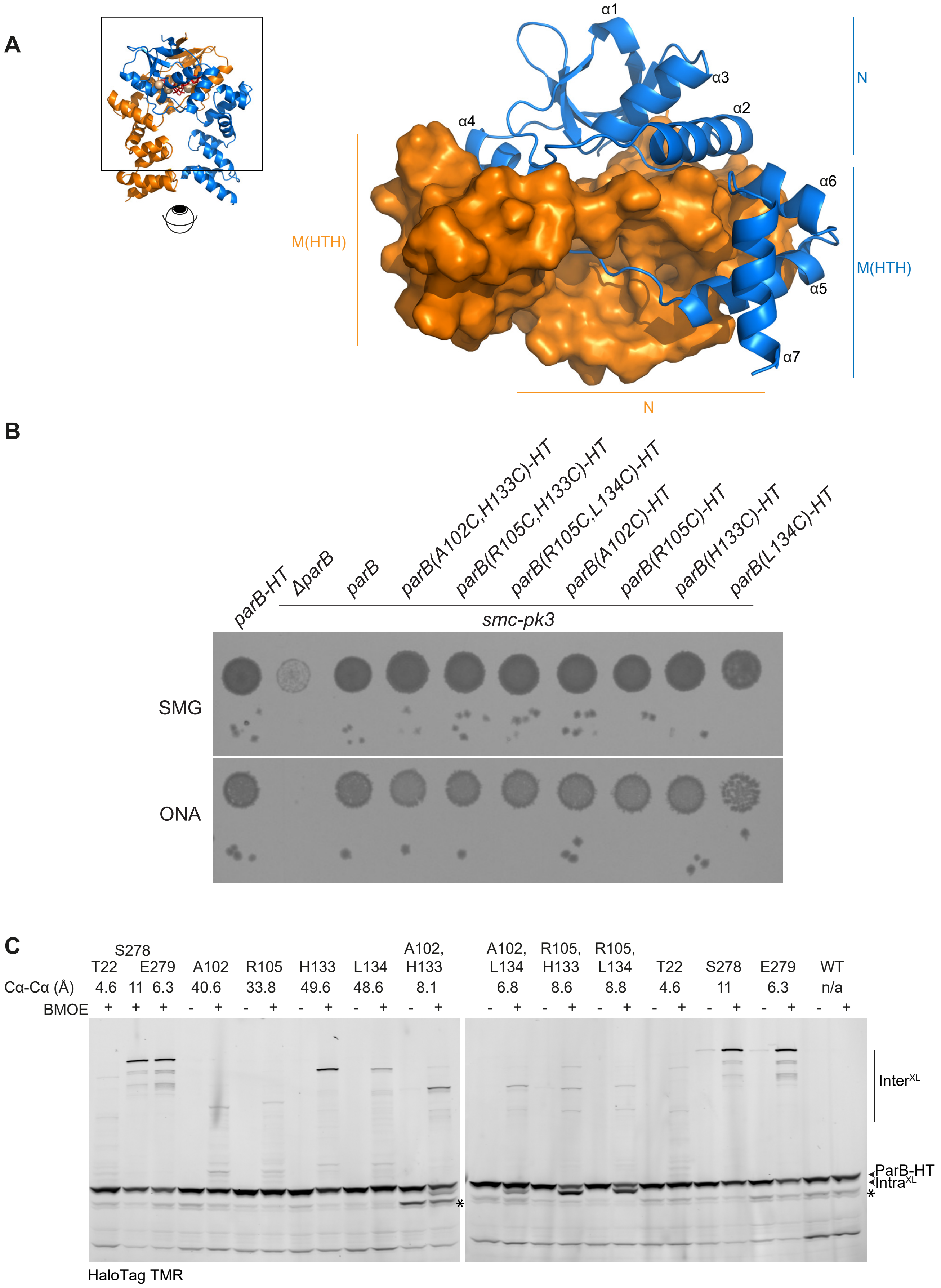

Fig. S1 Engineering cysteine pairs for ParB N-M interface cross-linking.
(A) ParB-CDP crystal structure (PDB: 6SDK) showing the tight interlocking between N and M domains from the two chains of a ParB dimer as viewed from the bottom.
(B) Growth assay by dilution spotting of strains harbouring different *parB* alleles combined with *smc-pk3*. 9^2 and 9^5-fold dilutions were spotted on minimal medium agar plates (SMG) and rich medium agar plates (ONA) and imaged after 24 and 16 hours, respectively.
(C) *In vivo* BMOE cross-linking of selected ParB cysteine mutants. Same as in Fig. 1A with additional strains and control reactions lacking BMOE. For reference, the Cα-Cα distances for pairs of residues on the ParB-CDP dimer structure (PDB: 6SDK) are given. Asterisks denote degradation products of ParB-HT.

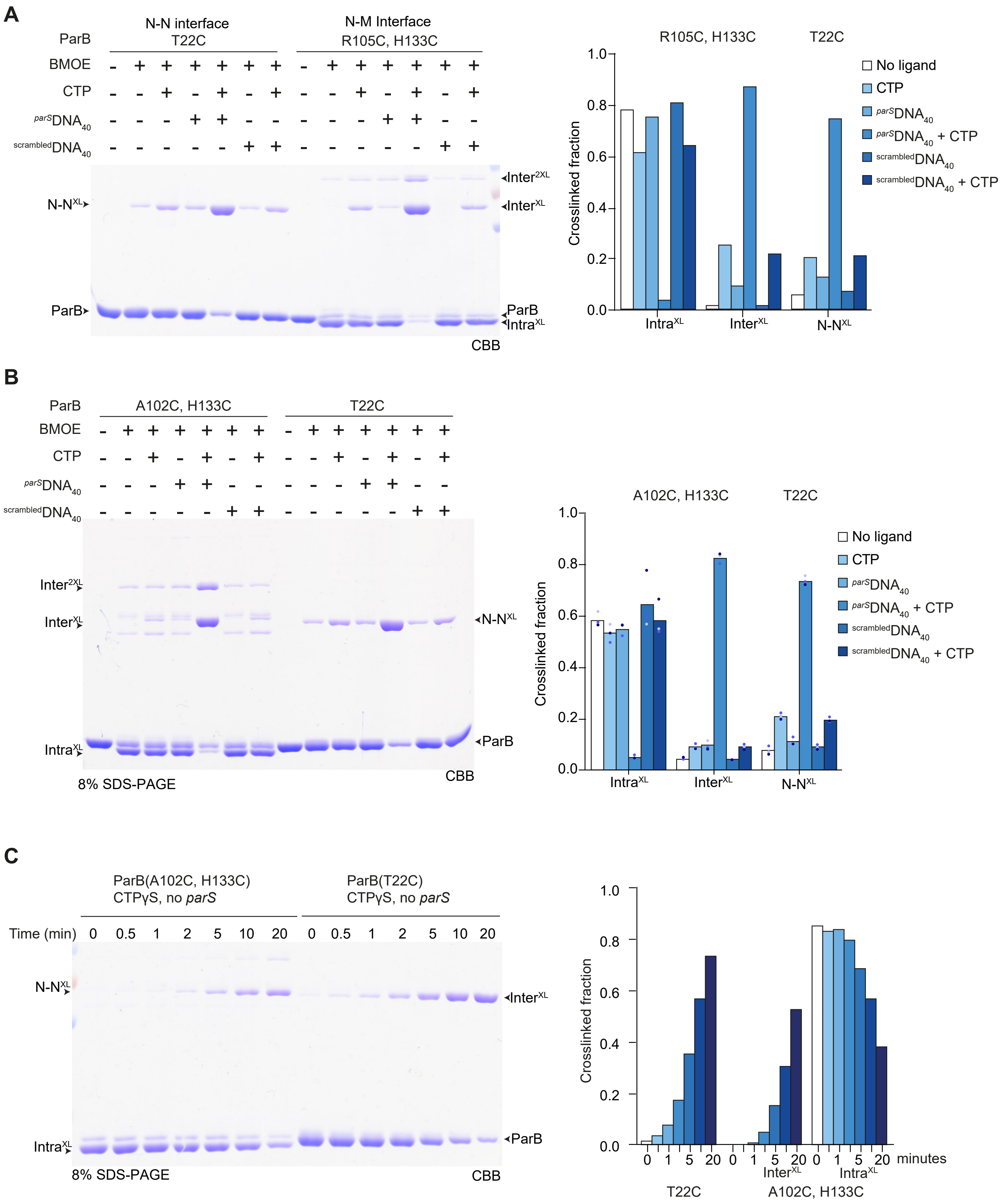

Fig. S2 ParB cysteine cross-linking of N-N and N-M interfaces.

(A) *In vitro* BMOE cross-linking of purified cysteine mutants ParB(T22C) and ParB(R105C, H133C). Cross-linked fractions were analysed by SDS-PAGE and CBB staining. Same gel picture as shown in Fig. 1B with estimation of cross-linking efficiencies from quantification of gel band intensities (right panel).

(B) *In vitro* BMOE cross-linking of purified mutants as in (A) but using ParB(T22C) and ParB(A102C, H133C). Quantification of cross-linking efficiency was done in three replicates. Mean and individual data points are shown.

(C) Time course of in vitro BMOE cross-linking of purified cysteine mutants ParB(A102C, H133C) and ParB(T22C) after addition of 1 mM CTPγS (in the absence of *parS* DNA). Quantification as in (A) (right panel).

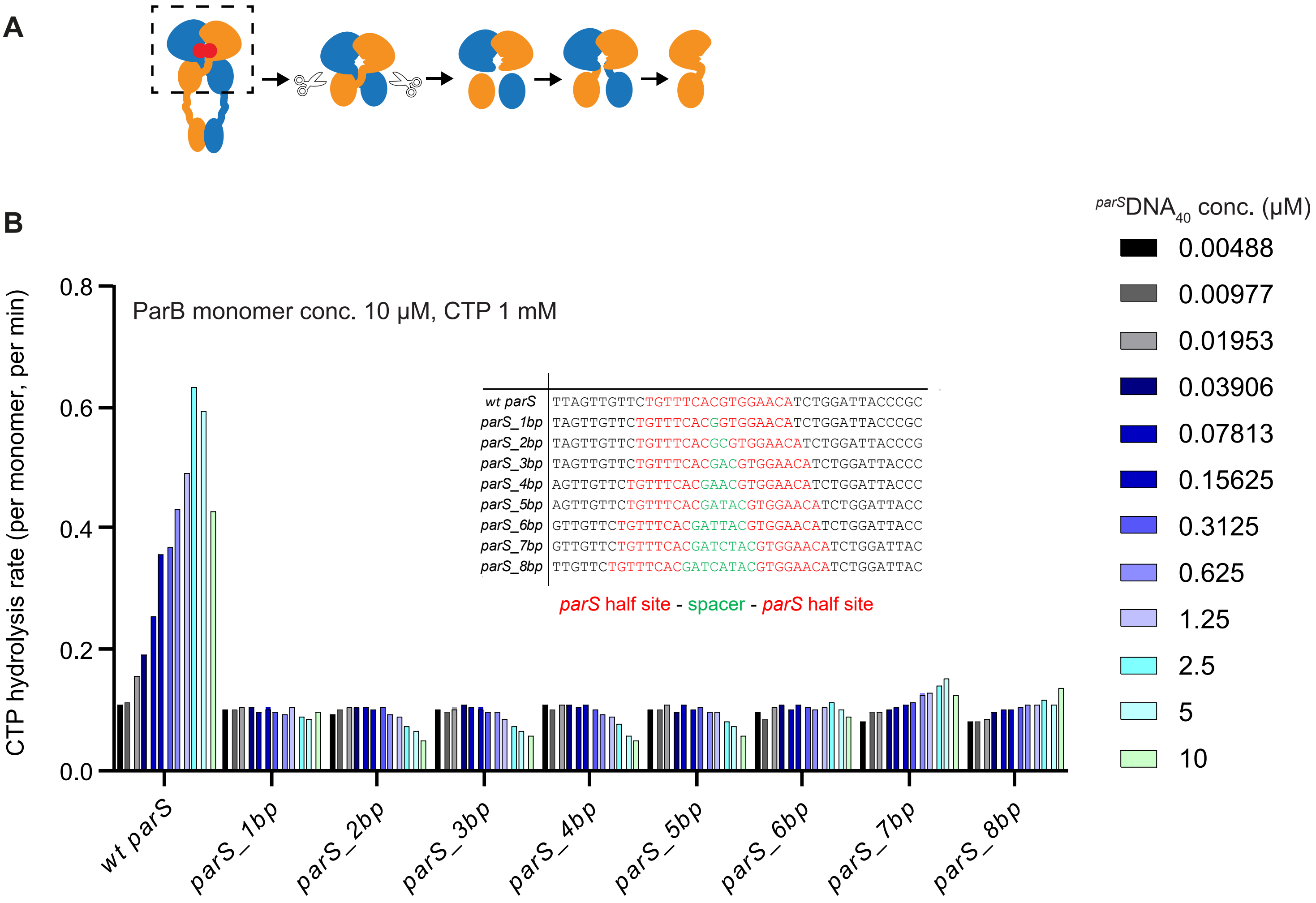

Fig. S3 Modulation of ParB CTP hydrolysis by altered *parS* DNA sequences.
(A) A model of the ParB^{Intra} monomer was created manually by linking residues 1-116 from chain A (PDB: 6SDK) to residues 117-218 of chain B.
(B) Rate of CTP hydrolysis by wild-type *B. subtilis* ParB measured by Malachite green assay. The rate was measured in response to the presence of different $^{parS}DNA_{40}$ sequences with increasing number of spacer base pairs between the two *parS* half sites. Final reaction contained 10 µM of ParB monomer, 1 mM CTP, and a serial dilution of altered *parS* DNA, in Mg2+ containing buffer.

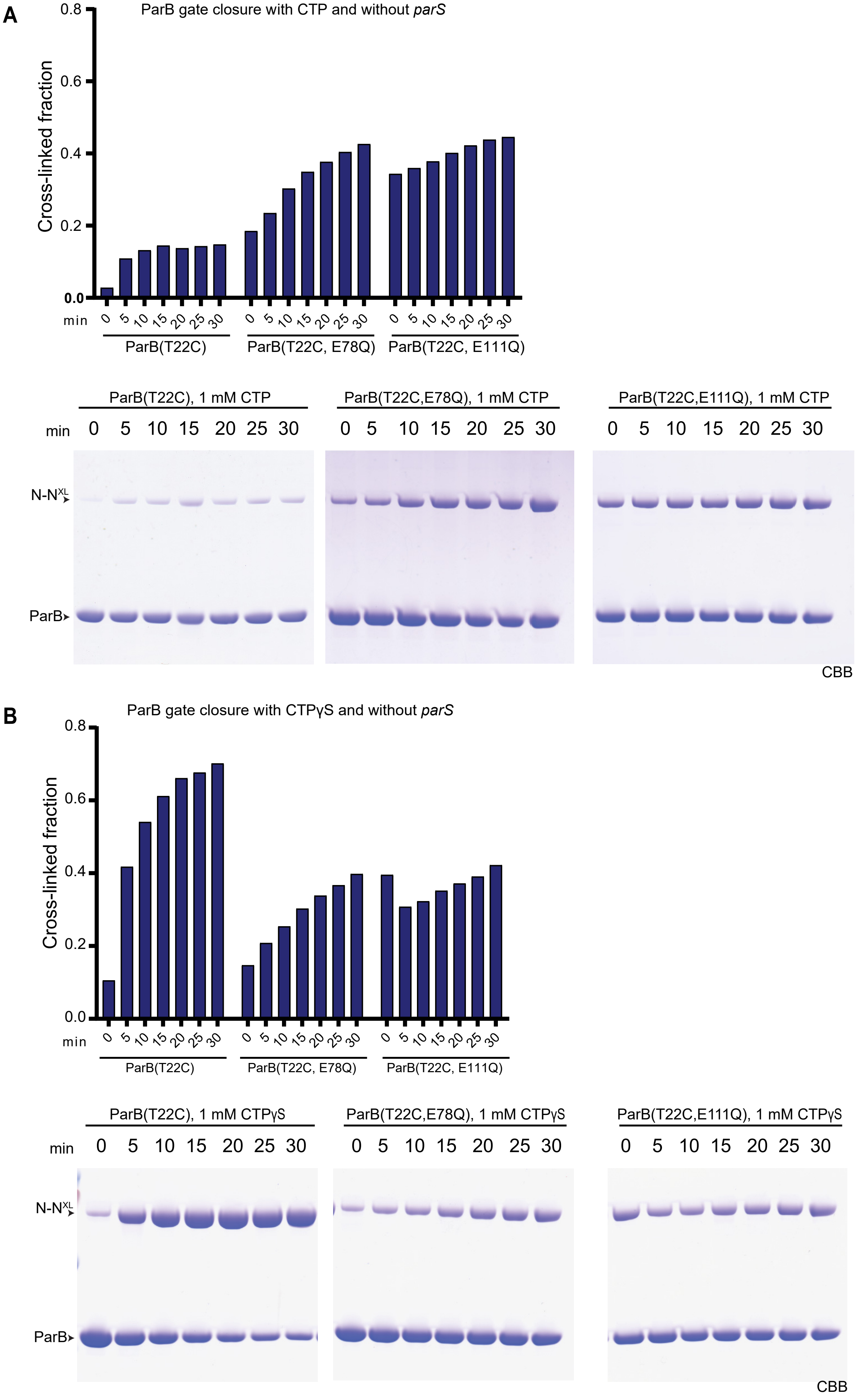

Fig. S4 ParB(EQ) cysteine cross-linking using T22C.

(A) Time course of *in vitro* BMOE cross-linking of ParB variants (10 μM), in the presence of CTP (1 mM) without *parS* DNA. Reaction is in Mg^{2+} containing buffer (see Materials and Methods). Quantification of bands was performed with ImageQuant (GE Healthcare).

(B) Same as in (A) but with CTPγS (1 mM).

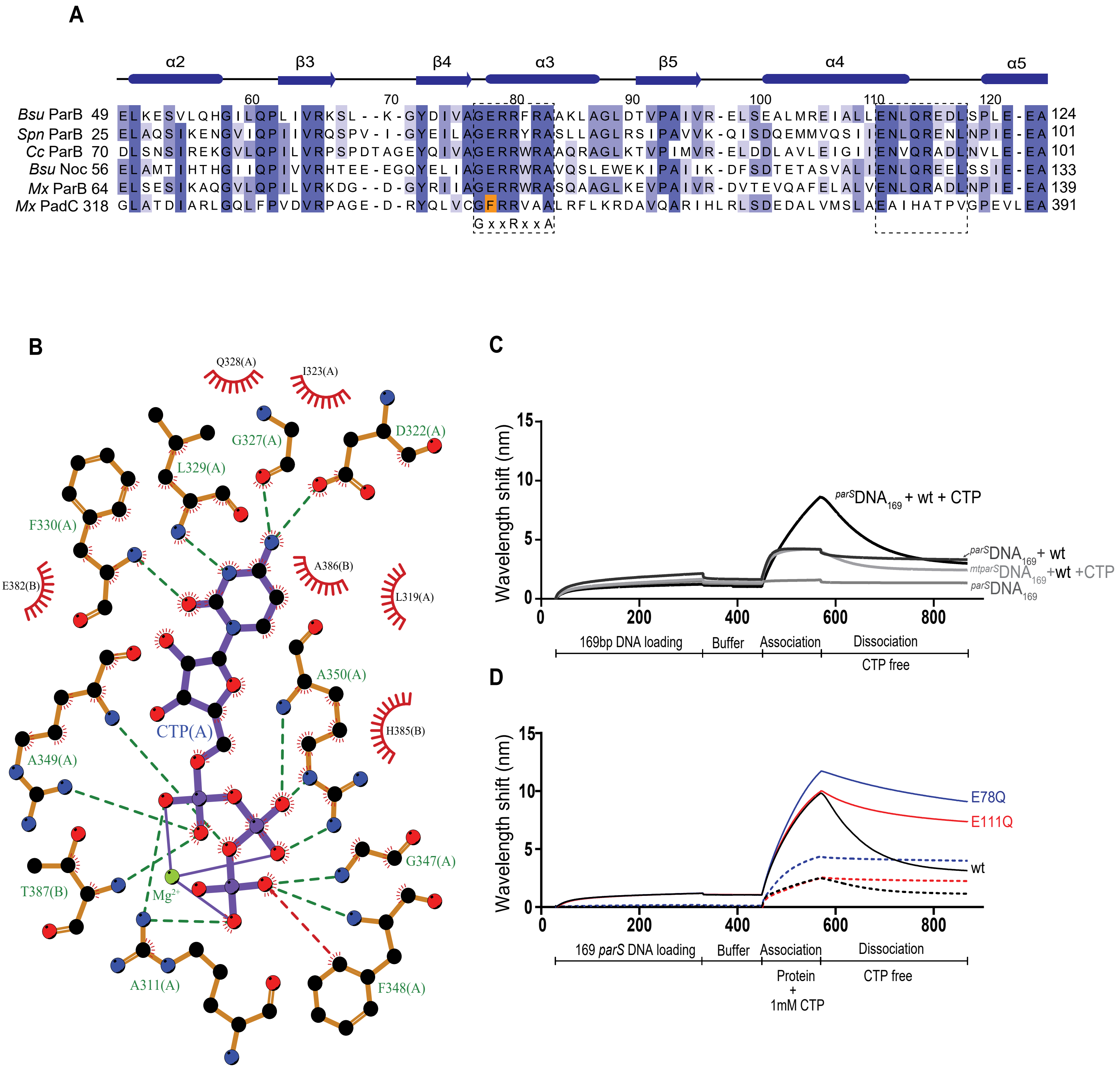

Fig. S5 The CTP binding pocket of ParB and ParB DNA loading.

(A) Alignment of central sequences of four bacterial ParB proteins (*Bsu, Bacillus subtilis; Spn, Streptococcus pneumoniae; Cc, Caulobacter crescentus; Mx, Myxococcus xanthus*), *Bsu* Noc and *Mx* PadC. The conserved GE$_{78}$RRxxA and E$_{111}$NLQRE motifs are marked by dashed boxes. The change of nucleotide (E78 to F348) in *Mx* PadC is marked in orange colour.

(B) 2D Protein-Ligand interaction map of PadC-CTP (PDB: 6RYK) showing residues from the two chains of PadC that contribute to the binding of one CTP molecule and one Mg^{2+}. Hydrogen bonds are represented by green dashed lines and hydrophobic interactions by red semi-circles (except for F348(A) extra red dashed line is added). The map was generated on LigPlot+ software.

(C) Biolayer interferometry assay measuring ParB loading efficiency on *parS* or *mtparS* 169 bp DNA in the presence or absence of CTP in the protein association phase. A negative control with no protein is also included (^{parS}DNA$_{169}$).

(D) Biolayer interferometry assay, same as in Fig. 3E, in addition to conditions where a single-end biotin labelled *parS* 169 bp DNA is used for ParB loading (dashed lines).

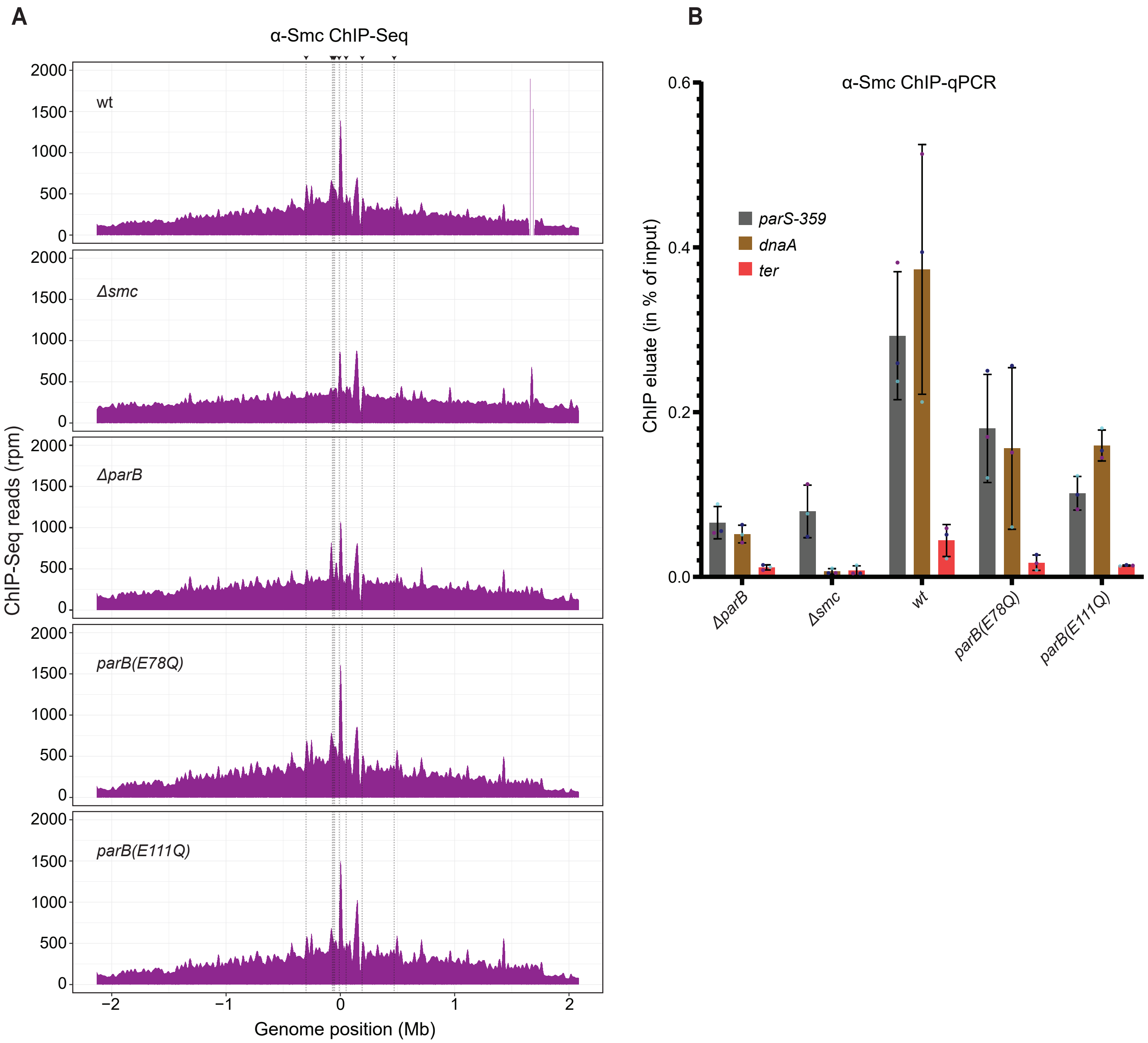

Fig. S6 anti-Smc Chromatin-immunoprecipitation.
(A) Chromatin-immunoprecipitation coupled to deep sequencing (ChIP-Seq) using α-Smc serum. Panels show genome wide distribution of Smc enrichment. Same conditions in Fig. 3C but with the addition of *Δsmc* strain.
(B) Chromatin-immunoprecipitation coupled to quantitative PCR (ChIP-qPCR) using α-Smc serum. Mean values and standard deviation from three repeat measurements are reported. Individual data points are shown as dots. Enrichment of Smc was tested at the three loci: *parS-359*, *dnaA*, and the replication terminus (*yocGH*).

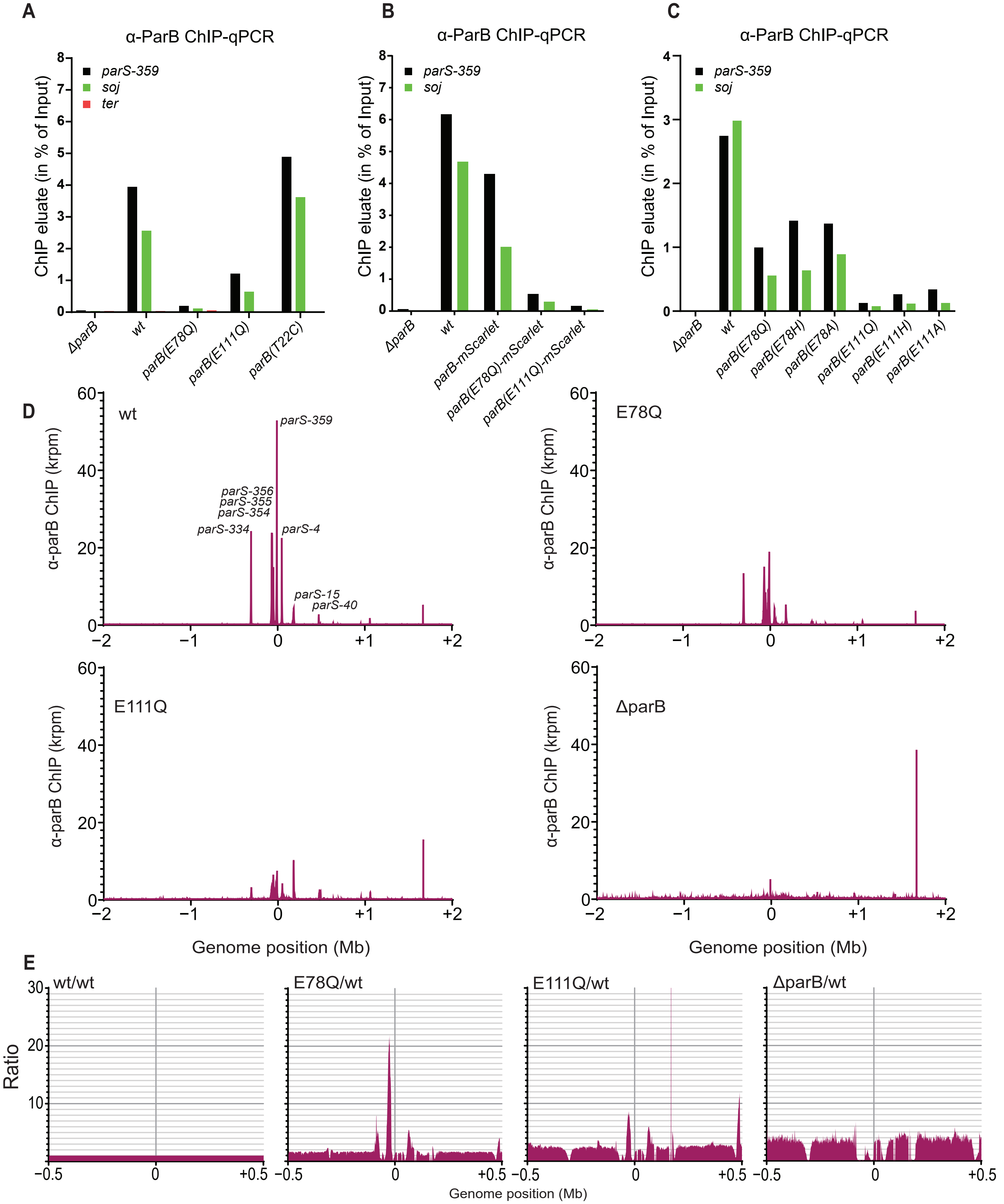

Fig. S7 anti-ParB Chromatin-immunoprecipitation.

(A) Chromatin-immunoprecipitation coupled to quantitative PCR (ChIP-qPCR) using α-ParB serum. Same as in Fig. 5A in addition to a strain carrying the *parB(T22C)* allele.

(B) Chromatin-immunoprecipitation coupled to quantitative PCR (ChIP-qPCR) using α-ParB serum for strains with mScarlet tagged ParB. As in (A).

(C) Chromatin-immunoprecipitation coupled to quantitative PCR (ChIP-qPCR) using α-ParB serum. Same as in Fig. 5A in addition to strains carrying histidine (H) and alanine (A) substitutions of E78 and E111 residues.

(D) Chromatin-immunoprecipitation coupled with deep sequencing (ChIP-Seq) using α-ParB serum. Same as in Fig. 5B but with the addition of a *ΔparB* strain and showing genome wide distribution. Of note, the peak at position +1.8 Mb represents enrichment at the *smc* gene presumed to be a contamination.

(E) Ratio plot of sequencing reads found in E78Q, E111Q, and *ΔparB* divided by read number in wild type. The region shown is 1 Mb wide surrounding the origin of replication. Peaks correspond to regions with higher enrichment of ParB(EQ) near parS sites (higher spreading efficiency).

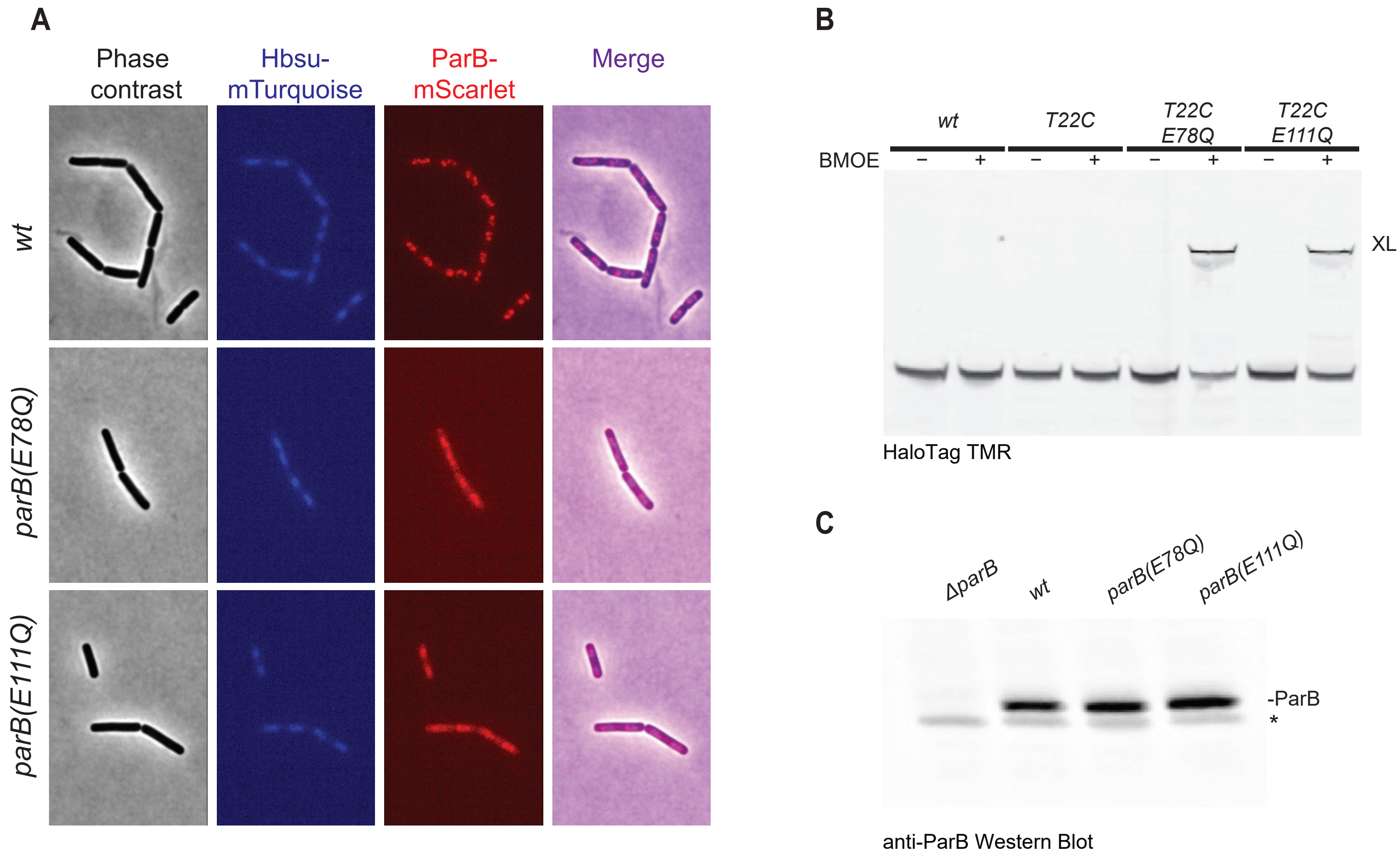

Fig. S8 Cellular expression and localization of ParB(EQ).
(A) Fluorescence microscopy imaging of *B. subtilis*: mScarlet tagged ParB (red) and mTurquoise tagged Hbsu (blue). Same as in Fig. 5C but cells were pre-incubated with 200 µg/ml of chloramphenicol for artificial nucleoid compaction for 15 minutes prior to imaging. The images show colocalization of ParB-mScarlet signal with nucleoids staining.
(B) Cysteine crosslinking control showing the absence of crosslinked ParB-HT variants in conditions with no added BMOE as judged by HaloTag-TMR labelling and in-gel fluorescence detection. ParB-HT variants harbor the T22C residues and the E78Q or E111Q mutation.
(C) Expression levels of ParB variants as judged by immuno-blotting using anti-ParB serum. The asterisk denotes a non-specific band that indicates uniform loading.

Table S1: Strains used in this study.

Identifier	Genotype	Source
BSG1001	1A700, trpC2	Gruber lab
BSG1007	1A700, Δsmc ftsY::ermB, trpC2	Gruber lab
BSG1050	1A700, ΔparB::kanR, trpC2	Gruber lab
BSG2203	1A700, parB-HaloTag::specR, trpC2	Gruber lab
BSG2968	1A700, ΔparA, parB::kanR, trpC2	Gruber lab
BSG3388	1A700, parB(T22C)-linker-HaloTag(C61V,C262A)::specR, trpC2	Gruber lab
BSG3397	1A700, parB(S278C)-linker-HaloTag(C61V,C262A)::specR, trpC2	Gruber lab
BSG3398	1A700, parB(E279C)-linker-HaloTag(C61V,C262A)::specR, trpC2	Gruber lab
BSG3945	1A700, smc-Pk3 ftsY::specR, ΔparA: SpnParB::kanR, trpC2	Gruber lab
BSG3949	1A700, smc-Pk3 ftsY::specR, recoded ParB::camR, trpC2	Gruber lab
BSG4050	1A700, parB(E111Q)::kanR, trpC2	This work
BSG4051	1A700, parB(E111H)::kanR, trpC2	This work
BSG4052	1A700, parB(E111A)::kanR, trpC2	This work
BSG4053	1A700, parB(E78Q)::kanR, trpC2	This work
BSG4054	1A700, parB(E78H)::kanR, trpC2	This work
BSG4055	1A700, parB(E78A)::kanR, trpC2	This work
BSG4056	1A700, smc-Pk3 ftsY::specR, parB(E111Q)::kanR, trpC2	This work
BSG4059	1A700, smc-Pk3 ftsY::specR, parB(E78Q)::kanR, trpC2	This work
BSG4113	1A700, parB(A102C)-linker-HaloTag(C61V,C262A)::specR, trpC2	This work
BSG4114	1A700, parB(R105C)-linker-HaloTag(C61V,C262A)::specR, trpC2	This work
BSG4115	1A700, parB(H133C)-linker-HaloTag(C61V,C262A)::specR, trpC2	This work
BSG4116	1A700, parB(L134C)-linker-HaloTag(C61V,C262A)::specR, trpC2	This work
BSG4117	1A700, parB(A102C, H133C)-linker-HaloTag(C61V,C262A)::specR, trpC2	This work
BSG4118	1A700, parB(A102C, L134C)-linker-HaloTag(C61V,C262A)::specR, trpC2	This work
BSG4119	1A700, parB(R105C, H133C)-linker-HaloTag(C61V,C262A)::specR, trpC2	This work
BSG4120	1A700, parB(R105C, L134C)-linker-HaloTag(C61V,C262A)::specR, trpC2	This work
BSG4140	1A700, parB(T22C, E78Q)-linker-HaloTag(C61V,C262A)::specR, trpC2	This work
BSG4141	1A700, parB(T22C, E111Q)-linker-HaloTag(C61V,C262A)::specR, trpC2	This work
BSG4156	1A700, parB(A102, H133C, S278C)-linker-HaloTag(C61V,C262A)::specR, trpC2	This work
BSG4356	1A700, Δsmc ftsY::ermB, parB(E111Q)::kanR, trpC2	This work
BSG4357	1A700, Δsmc ftsY::ermB, parB(E78Q)::kanR, trpC2	This work
BSG4666	1A700, parB-mScarlet::KanR, ΔcgeD::hbsU-mTorquois::CAT, trpC2	This work
BSG4667	1A700, parB(E111Q)-mScarlet::KanR, ΔcgeD::hbsU-mTorquois::CAT, trpC2	This work
BSG4668	1A700, parB(E78Q)-mScarlet::KanR, ΔcgeD::hbsU-mTorquois::CAT, trpC2	This work
BSG4710	1A700, parB(E78Q A102C H133C S278C)-linker-HaloTag(C61V,C262A)::specR, trpC2	This work
BSG4714	1A700, parB(R105C, H133C, S278C)-linker-HaloTag(C61V,C262A)::specR, trpC2	This work
BSG4716	1A700, parB(A102C E111Q H133C S278C)-linker-HaloTag(C61V,C262A)::specR, trpC2	This work
BSG4718	1A700, parB(T22C, E78Q, S278C)-linker-HaloTag(C61V,C262A)::specR, trpC2	This work
BSG4719	1A700, parB(T22C, E111Q, S278C)-linker-HaloTag(C61V,C262A)::specR, trpC2	This work
BSG4793	1A700, parB(E78Q,R105C,H133C,S278C)-linker-HaloTag(C61V,C262A)::specR, trpC2	This work
BSG4795	1A700, parB(R105C,E111Q,H133C,S278C)-linker-HaloTag(C61V,C262A)::specR, trpC2	This work

BSG4775	1A700, smc-Pk3 ftsY::tetR, parB(A102C,H133C)-linker-HaloTag(C61V,C262A)::specR, trpC2	This work
BSG4776	1A700, smc-Pk3 ftsY::tetR, parB(R105C,H133C)-linker-HaloTag(C61V,C262A)::specR, trpC2	This work
BSG4777	1A700, smc-Pk3 ftsY::tetR, parB(R105C,L134C)-linker-HaloTag(C61V,C262A)::specR, trpC2	This work
BSG4778	1A700, smc-Pk3 ftsY::tetR, parB(A102C)-linker-HaloTag(C61V,C262A)::specR, trpC2	This work
BSG4779	1A700, smc-Pk3 ftsY::tetR, parB(R105C)-linker-HaloTag(C61V,C262A)::specR, trpC2	This work
BSG4780	1A700, smc-Pk3 ftsY::tetR, parB(H133C)-linker-HaloTag(C61V,C262A)::specR, trpC2	This work
BSG4781	1A700, smc-Pk3 ftsY::tetR, parB(L134C)-linker-HaloTag(C61V,C262A)::specR, trpC2	This work
BSG4815	1A700, Δ8-parS, parB(T22C, E111Q, mtparS)-linker-HaloTag(C61V,C262A)::specR, smc::ermB, trpC2	This work
BSG4816	1A700, Δ8-parS, parB(T22C, E78Q, mtparS)-linker-HaloTag(C61V,C262A)::specR, smc::ermB, trpC2	This work
BSG4852	1A700, Δ8-parS, parB(mtparS)-linker-HaloTag(C61V,C262A)::specR, smc::ermB, trpC2	This work

Table S2: Primers used in this study.

Identifier	sequence 5'-3'	oligo	source
STG097	AAAAAGTGATTGCGGAGCAG	F *parS-359* qPCR	Gruber Lab
STG098	AGAACCGCATCTTTCACAGG	R *parS-359* qPCR	Gruber Lab
STG099	TCCATATCCTCGCTCCTACG	F in *YocGH* (*ter*) for qPCR	Gruber Lab
STG100	ATTCTGCTGATGTGCAATGG	R in *YocGH* (*ter*)for qPCR	Gruber Lab
STG199	GATCAATCGGGGAAAGTGTG	F downsream of DnaA for qPCR	Gruber Lab
STG200	GTAGGGCCTGTGGATTTGTG	R downsream of DnaA for qPCR	Gruber Lab
STG238	TTTCCCTGCGGATCAATATC	F in Soj for qPCR	Gruber Lab
STG239	TGACATCGTGGGAAAAATCA	R in Soj for qPCR	Gruber Lab
STI706	TTAGTTGTTCTGTTTCACGTGGAACATCTGGATTACCCGC	F 40 bp *parS*	Gruber Lab
STI707	GCGGGTAATCCAGATGTTCCACGTGAAACAGAACAACTAA	R 40 bp *parS*	Gruber Lab
STO514	TAGTTGTTCTGTTTCACGGTGGAACATCTGGATTACCCGC	F 40 bp parS359+G	This work
STO515	GCGGGTAATCCAGATGTTCCACCGTGAAACAGAACAACTA	R 40 bp parS359+G	This work
STO516	TAGTTGTTCTGTTTCACGCGTGGAACATCTGGATTACCCG	F 40 bp parS359+GC	This work
STO517	CGGGTAATCCAGATGTTCCACGCGTGAAACAGAACAACTA	R 40 bp parS359+GC	This work
STO518	TAGTTGTTCTGTTTCACGACGTGGAACATCTGGATTACCC	F 40 bp parS359+GAC	This work
STO519	GGGTAATCCAGATGTTCCACGTCGTGAAACAGAACAACTA	R 40 bp parS359+GAC	This work
STO520	AGTTGTTCTGTTTCACGAACGTGGAACATCTGGATTACCC	F 40 bp parS359+GAAC	This work
STO521	GGGTAATCCAGATGTTCCACGTTCGTGAAACAGAACAACT	R 40 bp parS359+GAAC	This work
STO522	AGTTGTTCTGTTTCACGATACGTGGAACATCTGGATTACC	F 40 bp parS359+GATAC	This work
STO523	GGTAATCCAGATGTTCCACGTATCGTGAAACAGAACAACT	R 40 bp parS359+GATAC	This work
STO524	GTTGTTCTGTTTCACGATTACGTGGAACATCTGGATTACC	F 40 bp parS359+GATTAC	This work
STO525	GGTAATCCAGATGTTCCACGTAATCGTGAAACAGAACAAC	R 40 bp parS359+GATTAC	This work
STO526	GTTGTTCTGTTTCACGATCTACGTGGAACATCTGGATTAC	F 40 bp parS359+GATCTAC	This work
STO527	GTAATCCAGATGTTCCACGTAGATCGTGAAACAGAACAAC	R 40 bp parS359+GATCTAC	This work
STO528	TTGTTCTGTTTCACGATCATACGTGGAACATCTGGATTAC	F 40 bp parS359+GATCATAC	This work
STO529	GTAATCCAGATGTTCCACGTATGATCGTGAAACAGAACAA	R 40 bp parS359+GATCATAC	This work
